# Advanced Hydrogel Functionalization Strategies for Burn-Related Wound Treatment

**DOI:** 10.34133/bmr.0406

**Published:** 2026-09-28

**Authors:** Md Lemon Hasan, Mohammad Faysal Al Mazid, Yeasin Khan, Al Basir, Akhmad Irhas Robby, Shihab Uddin, Khandoker Asiqur Rahaman, Yoon Ki Joung

**Affiliations:** ^1^ Biomaterials Research Center, Biomedical Research Division, Korea Institute of Science and Technology (KIST), Seoul 02792, Republic of Korea.; ^2^ Department of Surgery, College of Medicine, Yonsei University, Seoul 06273, Republic of Korea.; ^3^ Department of Chemistry, Kyung Hee University, Seoul 02447, Republic of Korea.; ^4^ Interdisciplinary Research Center for Advanced Materials, King Fahd University of Petroleum and Minerals, Dhahran 31261, Saudi Arabia.; ^5^ Interdisciplinary Research Center for Biosystems and Machines, King Fahd University of Petroleum & Minerals, Dhahran 31261, Saudi Arabia.; ^6^ Department of Bioengineering, King Fahd University of Petroleum & Minerals, Dhahran 31261, Saudi Arabia.; ^7^ Division of Bio-Medical Science & Technology, University of Science and Technology (UST), Daejeon 34113, Republic of Korea.; ^8^ KHU-KIST Department of Converging Science and Technology, Kyung Hee University, Seoul, Republic of Korea.

## Abstract

Burn injuries cause deep, extensive skin damage and are associated with high morbidity and mortality. Effective treatment goes beyond superficial wound coverage; it requires regenerative repair that progresses from deeper tissue layers to complete skin restoration. Because of extreme trauma and severity, the fastest treatment strategies play a key role in the treatment of burn injuries. Recently, hydrogel dressings have gained attention as promising wound care options because they maintain a moist environment, provide cooling and pain relief, support autolysis, and promote tissue regeneration. However, the lack of biological function and, consequently, slower tissue regeneration limit the applications of hydrogels. In recent years, collaboration between doctors, materials scientists, and biologists has substantially improved the biological functionality of hydrogels. Integrating bioactive agents, such as proteins, peptides, antimicrobial compounds, drugs, metal nanoparticles, cell-based vesicles, and extracellular matrix, enables the development of multifunctional, active burn wound dressings. This review highlights advances in the functionalization of hydrogel dressings for burn care, including fabrication methods, biological efficacy, and therapeutic mechanisms. The literature provides a comprehensive overview of the area where further advancement could be pursued.

## Introduction

Burn injury is generally referred to as an epidermal injury caused by fire, electricity, radiation, or chemicals. Millions of individuals around the globe suffer from burn injuries every year, and it is considered the most devastating form of trauma among the victims [1]. Severe burns often require prolonged hospitalization, complex surgical interventions, and intensive rehabilitation, which is often burdensome for low- and middle-income countries where specialized treatments and logistical support are limited [2]. In addition to the immediate threat to life, burn injuries frequently lead to chronic complications, including infection, excessive inflammation, hypertrophic scarring, and impaired tissue regeneration [3–5]. Therefore, advancing therapeutic approaches for rapid wound healing has become a major priority.

In recent times, advanced therapies such as skin grafting, antimicrobial creams, and negative-pressure wound therapy have improved treatment outcomes. However, they still pose several limitations, such as donor-site morbidity, high cost, and infection risk. As a result, the development of innovative biomaterials that actively promote tissue repair has become a major focus in burn care research [6–10]. Hydrogels have recently emerged as a promising biomaterial for burn treatment due to their unique physicochemical and biological properties [11]. Their 3-dimensional (3D) crosslinked polymeric networks can retain large amounts of water while maintaining structural integrity [12,13]. This enables them to mimic natural tissue by providing a moist environment, which is essential for metabolic activities. Additionally, the tunable mechanical properties of hydrogels readily adapt to irregular wound surfaces, making them particularly suitable for burn wound applications [11,14].

Despite these advantages, hydrogels often exhibit limitations regarding insufficient antimicrobial activity, low mechanical strength, and uncontrolled drug release. In recent times, researchers have developed various custom functionalization strategies to enhance the biological performance of hydrogel systems. Many bioactive molecules, nanoparticles, and drugs can be incorporated into the hydrogel structure for targeted therapeutic purposes [15]. For example, antimicrobial peptides and metal nanoparticles can be integrated into hydrogels to prevent bacterial infection, while cell-adhesive peptides and ECM proteins can improve cell attachment and tissue integration. Additionally, stimuli-responsive functionalization enables hydrogels to release therapeutic agents in response to environmental cues such as pH, temperature, reactive oxygen species (ROS), or enzymatic activity [16–18]. These advanced approaches significantly enhance the therapeutic efficacy of hydrogel systems and provide new opportunities for designing smart wound dressings. Given the rapid advances in biomaterial engineering and regenerative medicine, it is important to critically evaluate current progress in hydrogel functionalization strategies and their potential clinical translation.

Although several reviews have discussed hydrogel-based wound dressings and skin regeneration, most focus primarily on material composition, fabrication technologies, or general wound-healing applications. In contrast, we specifically focus on advanced hydrogel functionalization strategies for burn wound treatment. This review critically examines how different bioactive components address key pathological features of the burn microenvironment, including bacterial infection, oxidative stress, excessive inflammation, impaired angiogenesis, and extracellular matrix (ECM) disruption. Furthermore, we discuss mechanistic pathways and burn-depth-specific therapeutic requirements. We address the current translational limitations and emerging opportunities, including antibiofilm therapies, artificial intelligence (AI)-assisted biomaterial design, and personalized hydrogel systems. By integrating these perspectives, this review aims to provide a more comprehensive framework for the rational design and clinical translation of next-generation burn wound hydrogels.

## Pathophysiology of Burn Wound Healing

### Classification of burns (degree and depth)

Clinical burn classification primarily depends on the depth of tissue injury [1]. It helps predict spontaneous healing potential, infection risk, and the need for excision or grafting. Burns are mainly categorized into superficial (first-degree), partial-thickness (second-degree), full-thickness (third-degree), and fourth-degree based on depth, each with distinct healing traits [19]. Burn depth and healing potential are crucial for treatment and care, but clinical assessment alone can achieve only 60% to 75% accuracy. Currently, laser Doppler imaging (LDI) is the most reliable method for predicting wound outcomes [20]. However, burn wound depth significantly influences treatment and morbidity. A combination of clinical evaluation, LDI, and ICG video angiography currently offers the most accurate assessments to ensure the best results [21].

### Hemostasis

Hemostasis is a precisely controlled physiological process involving platelets, coagulation factors, and vessel wall components that quickly seals injured blood vessels while keeping normal blood flow [22]. The hemostatic response forms a hierarchically arranged structure with a core of fully activated platelets surrounded by an unstable shell of less activated platelets, where thrombin activity and fibrin formation primarily occur in the innermost core. This spatial and temporal organization results from different elements of the platelet-signaling network and agonist concentration gradients [23,24]. Besides stopping bleeding, the hemostatic system also regulates angiogenesis, with activated platelets forming a temporary barrier that becomes embedded in polymerized fibrin. Proteins produced by the hemostatic system help stabilize the clot, followed by endothelial cell growth and vessel repair, with the fibrin network serving as a scaffold for cell migration and tissue rebuilding [25]. Bleeding after a burn injury largely depends on the burn’s depth. Superficial and superficial partial-thickness burns typically cause minimal or no significant bleeding because many of the deeper dermal vessels remain intact. In contrast, deep partial-thickness, full-thickness, and fourth-degree burns may damage the dermal vascular plexus and underlying tissues, resulting in vascular disruption and possible clinically significant bleeding [21,22]. Unlike sharp traumatic wounds caused by vessel laceration, burn-related bleeding occurs alongside thermal tissue necrosis, microvascular injury, coagulation activation, endothelial dysfunction, and intense inflammation. This creates a unique wound environment that can influence healing and scar formation [23,24,26]. Therefore, hydrogel-based therapies should be selected and engineered according to the depth, severity, and healing requirements of the burn wound (Fig. 1).

**Fig. 1.**
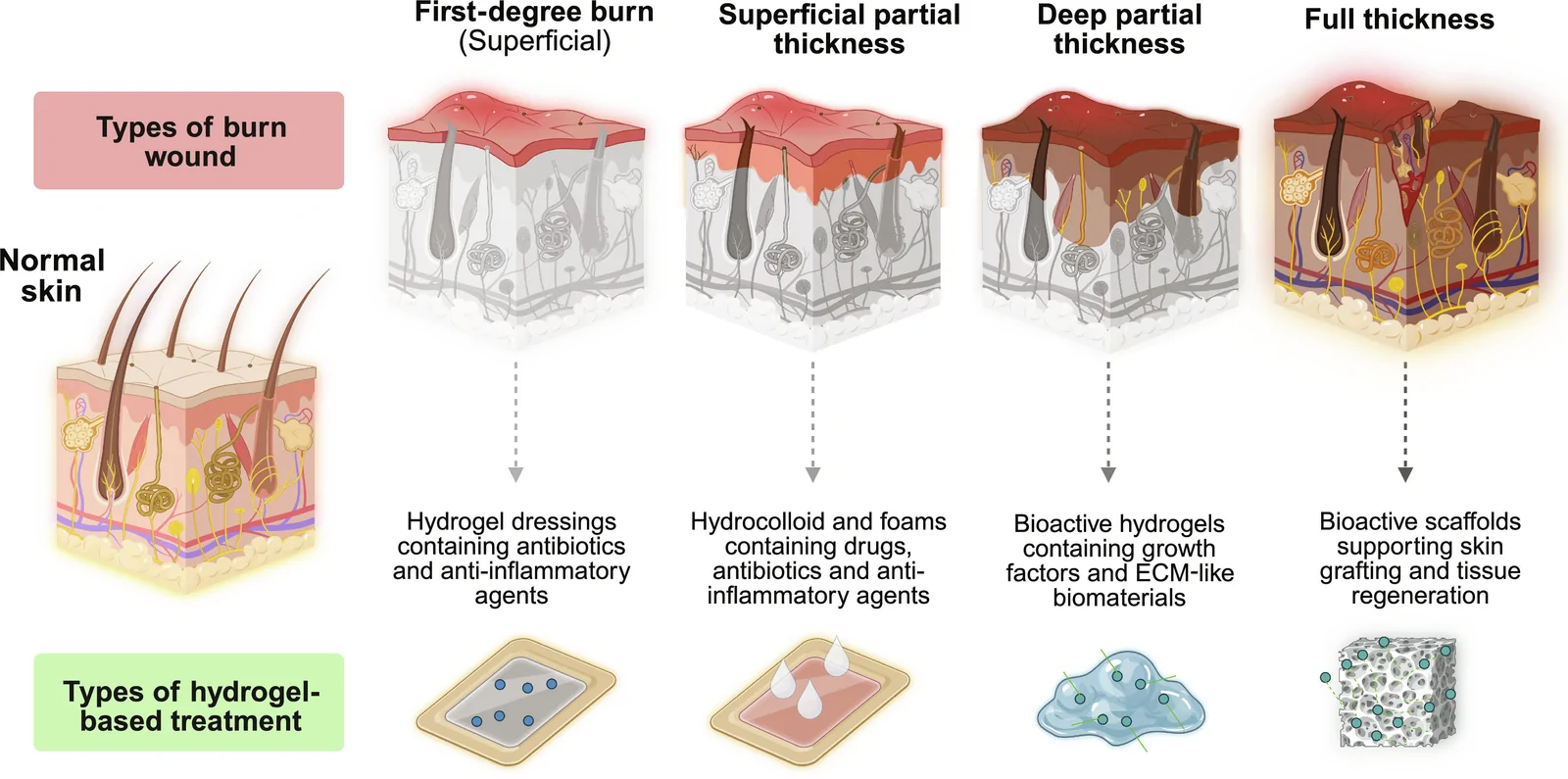
Schematic illustration of burn wound classification according to tissue depth and the corresponding hydrogel-based treatment strategies. The figure highlights first-degree (superficial), superficial partial-thickness, deep partial-thickness, and full-thickness burns, together with representative hydrogel systems ranging from conventional dressings to advanced bioactive scaffolds for tissue regeneration. The figure was created with Biorender.com.

Although hemostatic hydrogels quickly control bleeding and stabilize early wound conditions [27], their long-term impacts warrant careful evaluation. Excessive or prolonged coagulation may contribute to fibrin accumulation, persistent inflammatory signaling, fibroblast activation, fibrotic remodeling, and hypertrophic scar formation [28,29]. In burn injuries, where inflammation and dermal damage are already significant, hydrogel systems that coagulate rapidly should be designed to provide temporary hemostasis without promoting fibrotic signaling during later healing phases. Future hemostatic hydrogel designs should aim to stabilize clots immediately while allowing controlled breakdown, managing inflammation, and minimizing scar formation through tissue remodeling.

### Inflammation

Burn injuries initiate a complex inflammatory response that differs significantly from typical wound healing, characterized by prolonged and excessive immune activation both locally and systemically [26,30]. The local burn response shows persistent increases in immune cells, with immediate neutrophil surges and moderate rises in monocytes/macrophages and lymphocytes, especially in weeks 2 to 3 after the burn [26]. Macrophages are critical in post-burn immune dysfunction, driven by hyperactivity and increased production of pro-inflammatory mediators, which contribute to increased vulnerability to sepsis and multiple organ failure [31]. The inflammatory cascade involves complement activation, acute-phase proteins, pro- and anti-inflammatory mediators, and changes in lymphocyte activity, leading to both local and systemic pathology [30]. This extended inflammatory phase, marked by neutrophil surges, high levels of inflammatory cytokines, and limited immunosuppression, delays wound healing and raises the risk of complications [26,32]. Recent research shows that inflammation plays a dual role in wound healing, with its intensity determining whether outcomes favor regeneration or pathological scarring. Macrophages play a key role in this process, connecting inflammation and tissue repair by clearing debris, supporting stem cells, and promoting angiogenesis, but they can also drive chronic inflammation and excessive scarring if resolution fails [33]. The extent and duration of inflammation profoundly impact healing outcomes, with prolonged responses leading to increased scar formation and possibly preventing wound closure [28]. When inflammatory responses become dysregulated, wounds can turn chronic or progressively fibrotic, ultimately impairing tissue function [34]. This challenges the traditional view that inflammation is always beneficial for healing, as studies show that inflammation can delay healing and increase scarring, underscoring the need for a more nuanced understanding of controlled versus maladaptive inflammatory responses [35]. In severe burns, disruption of the epidermal and dermal barriers may increase the penetration and potential systemic absorption of topically applied therapeutic agents [36]. While hydrogel dressings primarily aim to support local healing by keeping the wound moist, protecting the wound bed, and enabling the gradual release of bioactive substances, their therapeutic cargo may also influence systemic inflammatory response syndrome, especially when used on extensive or highly vascularized burn wounds [18,37]. Consequently, designing topical hydrogel systems for severe burn treatment requires balancing effective local therapy with controlled drug release, minimizing systemic exposure, and carefully considering potential systemic immunomodulatory effects.

### Proliferation

Wound healing involves complex, coordinated phases, including inflammation, proliferation, and remodeling, with re-epithelialization being a key step in successful tissue repair [38,39]. During the proliferation phase, keratinocyte migration and proliferation promote epithelial closure, while fibroblasts deposit collagen-rich ECM, and endothelial cells support new blood vessel formation [38,40]. Growth factors (GFs), cytokines, and matrix metalloproteinases (MMPs) are essential regulators of these cellular activities [38]. However, healing can be hindered by elevated levels of inflammatory mediators, such as interleukin-1β (IL-1β), which increase MMP activity and degrade collagen [40]. Poor wound healing results from dysregulated inflammation, angiogenesis, matrix deposition, and cell recruitment, often linked to conditions such as diabetes, vascular disease, or aging [41]. Understanding these cellular and molecular mechanisms is vital for developing effective treatments for both acute and chronic wound care [39,41].

### Remodeling

Wound remodeling is a complex and lengthy process that can last from months to years after initial healing, involving the replacement of a temporary matrix with organized extracellular components to restore tensile strength [42]. In burn injuries, this process is especially vulnerable to pathological outcomes because of deep dermal destruction and ongoing inflammatory signals. Key cellular contributors include deep dermal fibroblasts, myofibroblasts, and fibrocytes, which influence scarring through profibrotic and antifibrotic signals [43]. Hypertrophic scarring affects up to 72% of burn patients and can lead to contractures, pain, and functional impairment. While the role of collagen in pathological scarring is well-understood, there are still significant gaps in knowledge regarding proteoglycan involvement in dermal fibrosis [44]. Recent advances are focusing on tissue-engineered matrices, autologous cell therapies, and predictive biomarkers to transition from reactive to regenerative treatment approaches [45].

### Major local and systemic complications associated with burn wounds

Burn injuries pose significant clinical challenges, with infection being a major cause of morbidity and mortality due to the loss of the protective skin barrier and systemic immune dysfunction [46,47]. Burn wounds initially remain sterile but then become colonized by gram-positive organisms, followed by gram-negative bacteria, with some populations at higher risk for drug-resistant organisms. Male gender, older age, full-thickness burns, and diabetes increase the risk of infection [46]. Beyond infection, severe burns result in pathological wound healing characterized by dermal fibrosis and excessive scarring, including hypertrophic scars and keloids that greatly impact quality of life. The systemic inflammatory response after burn injury promotes wound fibrosis and influences fibroblast behavior during healing [48]. Current burn treatments often fall short of ensuring optimal tissue regeneration and infection control, highlighting the need for innovative biomaterial strategies and dermal substitutes to improve patient outcomes [49]. Burn wounds often face complications beyond infection, including prolonged inflammation, oxidative stress, delayed re-epithelialization, impaired formation of new blood vessels, and chronic or fibrotic tissue development. We discuss those challenges and the mechanistic basis of the functionalized hydrogel system later.

## Hydrogel Systems for Burn Treatment

### Definition and fundamental properties

Hydrogel systems are 3D crosslinked polymeric networks capable of absorbing and retaining substantial amounts of water while maintaining their structural integrity [50]. Because of their high water content and resemblance to the ECM, hydrogels are promising biomaterials for burn wound treatment. The effectiveness of hydrogel dressings depends on their physicochemical and biological properties, including hydrophilicity, water retention, mechanical strength, oxygen permeability, biocompatibility, and biodegradability. These properties help hydrogels create a moist wound environment, allow gas exchange, offer mechanical protection, and support tissue healing. A thorough understanding of these key features is crucial for designing and improving hydrogel dressings for burn wounds.

### Hydrophilicity and water retention

Hydrophilic hydrogels are 3D polymeric networks containing water-attracting functional groups, such as hydroxyl (-OH), carboxyl (-COOH), amino (-NH_2_), and amide (-CONH_2_) groups, which enable them to absorb and retain large amounts of water within their crosslinked structure [50]. In the context of burn wound treatment, this hydrophilic nature allows hydrogel dressings to maintain a hydrated wound interface, reduce tissue dehydration, facilitate nutrient and oxygen diffusion, and support keratinocyte migration and epithelial regeneration [11,18]. Hydrogel hydrophilicity and hydration behavior can be characterized using water contact angle, swelling ratio, equilibrium water content, and water uptake capacity, whereas water vapor transmission rate provides information about moisture transport through the dressing [50–52].

Hydrogels represent an exciting development in burn wound care, providing better options than traditional dry dressings. These 3D polymer networks imitate the natural ECM and retain high water content, creating an ideal moist environment that promotes epithelial migration and reduces pain [11]. In burn first aid, hydrogels serve as effective alternatives to running water, especially during mass-casualty events or when clean water is scarce, providing cooling through heat loss via evaporation [53]. Unlike traditional dermal templates with simpler compositions, functional hydrogels offer improved properties, such as biodegradability, adhesiveness, antimicrobial effects, and support for angiogenesis, while adapting to irregular wound shapes and penetrating deeper tissue layers [37]. These networks enable controlled drug delivery and cell migration, although further clinical testing and manufacturing improvements are needed for wider use [18].

### Mechanical properties and elasticity

Natural and synthetic hydrogels can be engineered to exhibit adjustable mechanical and transport properties via different fabrication techniques. Natural hydrogels offer biocompatibility but have limited mechanical strength, while synthetic hydrogels allow precise customization for specific biomedical uses, including wound healing [54]. The mechanical properties of hydrogels are highly adjustable using various crosslinking methods, with poly(vinyl alcohol) (PVA) hydrogels showing great versatility; tensile strength can be varied from 50 kPa to 15 MPa, and toughness from 0.0167 to 150 MJ/m^3^ through ion-based modifications via the Hofmeister effect [55,56]. Physical crosslinking mechanisms create dynamic and reversible bonds that provide self-healing, tissue adhesion, and adaptability to complex wound shapes [57]. Unlike traditional dry dressings that need frequent changes and cause patient discomfort, hydrogels keep moisture balanced and imitate the natural ECM, leading to better wound-healing results [11].

### Oxygen permeability

Oxygen permeability is a crucial characteristic of hydrogel dressings, indicating their capacity to allow oxygen to pass from the external environment to the wound site while maintaining a moist microenvironment [11,14]. In burn wounds, this property directly influences oxygen management, as proper oxygen exchange is essential for cellular metabolism, collagen production, new blood vessel formation, and antimicrobial defense [58]. Recent research highlights the vital role of oxygen management in burn wound healing and in the development of hydrogel dressings. Adequate oxygen supply supports essential repair processes such as collagen production, blood vessel formation, and antimicrobial defense, while hypoxia hampers healing [58]. In addition to passive oxygen diffusion, oxygen-releasing hydrogel systems have been created as active platforms for managing oxygen in hypoxic burn wounds. Oxygen-releasing hydrogel systems have shown effectiveness in enhancing endothelial cell survival and tube formation under low-oxygen conditions, while reducing inflammation and promoting blood vessel growth and collagen deposition in burn models [58]. For superficial and normally vascularized burn wounds, passive oxygen diffusion through oxygen-permeable hydrogel dressings may be sufficient to support standard re-epithelialization and tissue repair [18]. However, in deep partial-thickness and full-thickness burns, vascular destruction, impaired perfusion, and localized hypoxia can reduce oxygen availability at the wound site [19,21]. Under these conditions, specialized oxygen-delivery approaches, including oxygen-generating or oxygen-releasing hydrogel systems, may provide additional benefits by enhancing local oxygen availability and supporting angiogenesis, collagen deposition, and tissue regeneration. Therefore, conventional oxygen permeability is mainly relevant for maintaining physiological oxygen exchange in less severe wounds, whereas active oxygen-delivery systems may be more suitable for severe, hypoxic, or poorly vascularized burn wounds. Hyperbaric oxygen therapy should be distinguished from these hydrogel-based approaches because it is a separate clinical modality involving systemic exposure to oxygen at elevated pressure, whereas oxygen-generating and oxygen-releasing hydrogels provide localized oxygen delivery at the wound interface [58,59]. Despite encouraging preclinical results, hydrogel-based wound dressings still need more clinical testing and manufacturing improvements for broader use [18].

### Biocompatibility and biodegradability

Natural hydrogels offer biocompatibility and biodegradability suitable for wound healing but lack mechanical strength, while synthetic hydrogels provide customizable properties but may compromise bioactivity [54]. Functional hydrogels offer advantages over traditional dermal templates, including high water content, biodegradability, adhesiveness, and antimicrobial activity, enabling better wound conformity and deeper tissue penetration [37]. These hydrogels create moist wound environments, promote cellular migration, and provide superior controlled drug delivery compared with conventional dressings [18]. Recent advances include multifunctional hydrogels incorporating selenium, silver, and copper nanopowders with natural bioactive compounds, achieving high moisture content (~90%) and excellent cell viability (up to ~160% of the control) while maintaining thermal stability and controlled swelling properties [60]. However, extensive clinical trials are still needed for widespread clinical use.

## Types of Hydrogels

Hydrogels are classified by criteria such as material composition, fabrication method, and delivery format. To avoid overlap among these systems, this review uses material composition as the primary classification framework and categorizes burn wound hydrogels as natural polymer-based, synthetic polymer-based, or hybrid/composite. Injectable, in situ-forming, implantable, patch, and 3D-printed hydrogels are treated as fabrication or delivery methods rather than as independent material classes, as they can be produced from natural, synthetic, or hybrid polymers. This approach clarifies the organization by distinguishing the hydrogel’s material composition from its fabrication or delivery process. These hydrogel classifications, their properties, and potential applications are summarized in Table 1.

**Table 1.** Types of hydrogels used in burn wound healing

Polymer source	Representative polymers	Key advantages	Limitations	Delivery mode	Ref.
Natural	• Alginate • Chitosan • Collagen • Gelatin • Hyaluronic acid (HA)	• High biocompatibility • Intrinsic bioactivity • Mimics ECM • Promotes cell adhesion	• Weak mechanical strength • Batch-to-batch inconsistency • Rapid degradation rate	**Injectable/In situ forming:**	
				• Chitosan-dextran hydrogel	[64]
				• Oxidized alginate-chitosan-quercetin hydrogel	[218]
				• Adipic acid dihydrazide-collagen hydrogel	[219]
				• HA-Konjac glucomannan	[220]
				• Gelatin-resveratrol-Histatin 1 hydrogel	[78]
				**Implantable/patch:**	
				• Alginate-gelatin/ZIF8	[81]
				• Keratin-chitosan/n-ZnO	[221]
				• Collagen-gelatin/transglutaminase hydrogel	[80]
				• Curcumin/carboxymethyl chitosan-thione	[222]
				• Gelatin-oxPullulan/black currant extract	[223]
				**3D-printed:**	
				• Gelatin-alginate/dermal fibroblast	[65]
				• Gelatin methacrylate-gelatin gallic acid hydrogel	[224]
				• Alginate-chitosan/Bromelain/Aloe vera	[225]
				• Gelatin-alginate/Bioactive borate glass	[85]
				• Chitosan methacrylate/Lidocaine HCl/Levofloxacin	[16]
Synthetic	• Polyacrylamide (PAM/PAAm) • Polyethylene glycol (PEG) • Polyvinyl alcohol (PVA) • Poloxamer (ex: Pluronic) • Poly(2-hydroxyethyl methacrylate) (PHEMA)	• High versatility • Tunable mechanical properties • Tunable swelling • High stability	• Lack of intrinsic bioactivity • Poor cellular adhesion • Lack of a natural degradation pathway	**Injectable/In situ forming:**	
				• PAAm/PDA/Vit. C	[226]
				• Poloxamer/Camelina lipid droplets-hFGF2	[67]
				• Pluronic F127/AgNP	[74]
				• PNIPAAM/Cellulose nanocrystal	[75]
				• PVA/Silk sericin/Vancomycin/Gentamicin	[227]
				**Implantable/patch:**	
				• PEG-dendritic macromonomer	[228]
				• PAAm/Calendula extract	[68]
				• PVA/Gentamicin	[229]
				• hMSC/hFDM/PVA	[230]
				• PMVEMA-PEG/Ibuprofen	[217]
				**3D-printed:**	
				• pHEMA-PEGDA/AgNP	[231]
				• PLGA/PEGylated Arg-Gly-Asp tripeptide (RGD)	[89]
				• PEO/Guar gum/Lawsone	[232]
				• NPR-patterned polycaprolactone (PCL)	[233]
				• PVA/Ciproflaxin-doped halloysite nanotube	[234]
Hybrid/Composite	Combining natural with synthetic polymers, nanomaterials, or inorganic materials	• High mechanical strength • Improved swelling • Antimicrobial activity • Dynamic responsiveness	• Complex and costly fabrication • Difficult to scale up • Encounter cytotoxicity from embedded inorganic nanomaterials	**Injectable/In situ forming:**	
				• Thiolated HA-PEGDA-CPR1P hydrogel	[70]
				• Hyperbranched PEG-HA-RGD peptide	[79]
				• Gelatin-polyglyceryl stearate/GO	[235]
				• Methylcellulose/Ag oxide	[236]
				• Aminated gelatin-HA/Fe (III)	[77]
				**Implantable/Patch:**	
				• Chitosan-glycerophosphate/Cu_3_SnS_4_	[237]
				• PLL-PGA/Curcumin/Mg-polyphenol	[163]
				• Ca-alginate/PNIPAAm	[238]
				• PAAm-Chitosan/PVA-chitosan double layer	[239]
				• PVA-PET/Melia azedarach extract	[240]
				**3D-printed:**	
				• GelMA-PLGA/concentrated growth factor	[241]
				• Alginate-PNIPAAm-methyl cellulose	[242]
				• Diatomite/GelMA	[72]
				• Alginate-dopamine/Cu^2+^/Ca^2+^	[243]
				• GelMA-alginate/Dexamethasone/Au nanorods	[244]

### Classification based on material composition

#### Natural polymer-based hydrogels

Natural polymer-based hydrogels are promising biomaterials for wound healing, especially in burn care, because they mimic the ECM and support key healing processes. These materials, such as chitosan, collagen, alginate, gelatin, and hyaluronic acid (HA), offer better biodegradability and biocompatibility than synthetic options [61–63]. For burn treatment, functional hydrogels outperform traditional dermal templates by fitting irregular wound shapes, retaining moisture, supporting cell migration, and enabling controlled drug release [37]. For example, injectable chitosan-dextran (Dex) hydrogels can rapidly conform to irregular burn injuries, while 3D-printed gelatin-alginate dressings offer efficient hydration activity [64,65]. Despite these advantages, natural hydrogels face distinct disadvantages, such as weak mechanical strength, structural instability, and rapid degradation rates. These limitations are often mitigated through crosslinking or by blending them with synthetic polymers to form hybrid systems [61,63].

#### Synthetic polymer-based hydrogels

Synthetic polymers such as polyethylene glycol (PEG), PVA, polycaprolactone (PCL), and poly(lactic-co-glycolic acid) (PLGA) offer customizable physicochemical properties and enhanced therapeutic functions for advanced wound dressings, with modifications such as crosslinking and blending further enhancing their effectiveness [66]. While natural hydrogels suffer from mechanical weakness and batch-to-batch inconsistency, synthetic hydrogels enable precise control over physical and chemical properties through advanced polymer chemistry. This versatility has been demonstrated through applications such as Poloxam hydrogels for fast gelation and controlled drug release, and PAAm-Calendula extract patches for high-mechanical-strength dressings with excellent tissue regeneration [67,68]. However, the primary limitation of synthetic hydrogels is that their customizable mechanical properties often come at the expense of intrinsic bioactivity and biocompatibility. Consequently, clinical implementation still requires further biological validation [14,18].

#### Hybrid/composite hydrogels

Hybrid and composite hydrogels are an emerging approach to tackling the multifactorial challenges of burn wound healing by combining natural and synthetic polymers with functional nanomaterials and bioactive agents. These systems summarize the optimal traits of both sources, which surpass the limitations of traditional hydrogels by providing the high mechanical strength of synthetic polymers alongside the innate bioactivity, antimicrobial properties, and dynamic responsiveness required in complex wound environments [69]. Zhang et al. designed an in situ-forming HA-PEGDA-CPR1P hydrogel that demonstrated rapid gelation in wound site and tunable mechanical properties owing to the PEGDA crosslinking, with enhanced vascular endothelial growth factor (VEGF) recruitment and angiogenesis due to HA and CPR1P [70]. It is also reported that incorporating inorganic materials into a hydrogel matrix to fabricate composite hydrogels can address multiple wound-healing needs, including hydration, infection prevention, and inflammation control [63,71]. For instance, Ma et al. [72] incorporated natural diatomite microparticles into the GelMA hydrogel to form a bioactive composite ink for 3D-printed wound dressing, where the presence of diatomite effectively enhanced the water absorption capacity and up-regulated the expression of angiogenesis-related genes. While highly effective, these smart dressing platforms represent a more complex manufacturing challenge regarding scalability and regulatory approval compared to their monolithic counterparts [14].

### Classification based on fabrication and delivery formats

#### Injectable and in situ-forming hydrogels

Injectable and in situ-forming hydrogels provide notable benefits for burn wound treatment by conforming to irregular wound shapes and enabling minimally invasive delivery [37,73]. These systems form gels in place through various mechanisms, including temperature-triggered gelation, as shown by Pluronic F127/AgNP and PNIPAAm/Cellulose nanocrystal hydrogels that undergo temperature-responsive gelation at body temperature owing to their lower critical solution temperature properties [74–76]. One of the major advancements is the creation of a multifunctional injectable aminated alginate-oxidized-HA-catechol-Fe^3+^ coordination hydrogel that offers self-healing, shape adaptability, strong tissue adhesion properties, as well as excellent antibacterial activity and oxidative stress protection, demonstrating ideal therapeutic efficacy for dynamic burn wound healing [77]. Moreover, injectable hydrogels can be integrated with biological materials (e.g., peptides, proteins, and enzymes) to address key burn complications by supporting re-epithelialization and neoangiogenesis and reducing inflammatory responses. Zheng et al. utilized histatin-1 (His-1) loaded in a supramolecular host–guest gelatin hydrogel to suppress inflammation and promote vascularization at burn wound sites, while Dong et al. incorporated short RGD peptide into the hyperbranched PEGDA hydrogel to promote cell adhesion, enhance cell proliferation, and increase the paracrine activity of angiogenesis [78,79]

#### Implantable and patch hydrogels

Implantable and patch hydrogels offer superior structural and functional control, crucial for managing severe thermal injuries, while injectable hydrogel systems excel at filling deep, irregular wound voids [53]. Basso et al. [80] reported the structural and practical differences of a collagen-gelatin/transglutaminase hydrogel patch by controlling the degree of crosslinking, wherein low crosslinking concentration yielded highly swollen and softer networks suitable for drug delivery, while high crosslinking concentration produced a stiffer structure ideal for load-bearing implantable dressing. These platforms provide an immediate physical barrier that prevents exogenous infection triggered by the unpredictable gelation kinetics or potential leakage of injectable formulations. Furthermore, the defined macroarchitecture of a patch enables the integration of prealigned topographical features that guide rapid cellular infiltration and re-epithelialization. The capacity for asymmetric design complements this structural integrity. For example, a hydrogel patch can be engineered with the addition of ZIF-8 nanoparticles to regulate the water absorption rate because their presence in the hydrogel matrix hinders water absorption by filling gaps in the structure [81]. Ultimately, the use of implantable or patch hydrogels ensures mechanical reliability and predictable degradation, which are essential for the rigorous safety demands of clinical translation in burn care.

#### 3D-printed hydrogels

3D printing technology has become a transformative approach for creating advanced burn dressings with spatially programmed architectures that surpass traditional casting methods. This manufacturing process enables precise control over porosity, gradient structures, and patient-specific geometries, which are vital for improving fluid management, oxygen delivery, and mechanical flexibility in burn care [82,83]. Different 3D printing techniques, including stereolithography, extrusion printing, and bioprinting, can produce customized dressings using biocompatible materials such as synthetic polymers and natural hydrogels [83,84]. Studies show that 3D-printed hydrogel dressings enhance wound healing, reduce pain and inflammation, and improve functional outcomes compared with conventional methods [83]. For instance, 3D-printed gelatin-alginate dressings have demonstrated improved wound closure, hair follicle regeneration, and nontraumatic removal compared to nonprinted hydrogels of similar composition [65]. A study conducted by Fayyazbakhsh et al. also highlighted the potential features of 3D-printed wound dressings containing bioactive borate glass (BBG), in which the 3D-printed porous surface supports nonadhesive contact and increases the available surface for wound-dressing interactions compared to nonprinted dressings. The presence of BBG further controls the release of water and therapeutic ions and improves dressing stability at the wound site [85]. This technology provides a design approach that expands the potential designs for burn dressings rather than representing a separate material category [82].

## Functionalization Strategies of Hydrogel for Burn Treatment

Hydrogels, used as biomaterials, provide a moist, biocompatible, and ECM-like environment that supports cell growth and protects the wound. Additionally, hydrogel matrix systems serve as versatile platforms for incorporating and delivering bioactive agents, including drugs, proteins, peptides, cells, extracellular vesicles, and nanoparticles. When functionalized with these bioactive components, hydrogels act as symbiotic systems that combine the structural advantages of the hydrogel matrix with the biological activity of the incorporated agents, as illustrated in Fig. 2. Importantly, the selection of hydrogel functionalization strategies should be guided by burn depth and the corresponding biological requirements of the wound [1,46]. Superficial and partial-thickness burns generally benefit from hydrogels functionalized with antimicrobial, antioxidant, and anti-inflammatory agents, which facilitate rapid epithelial recovery while maintaining a moist wound environment. In contrast, deep partial-thickness burns often require additional pro-regenerative functionalities, including angiogenic peptides, GFs, extracellular vesicles, and oxygen-generating systems, to overcome impaired vascularization and extensive tissue damage [18]. For full-thickness and fourth-degree burns, where dermal architecture, skin appendages, and vascular networks are severely compromised, advanced biofunctionalized hydrogels incorporating ECM-derived materials, stem cells, exosomes, or multifunctional composite systems may provide bioactive scaffolds that support tissue regeneration, vascular reconstruction, and scar reduction. These advanced hydrogel functionalization strategies promise to yield next-generation biomaterials capable of meeting the distinct therapeutic needs of different burn severities.

**Fig. 2.**
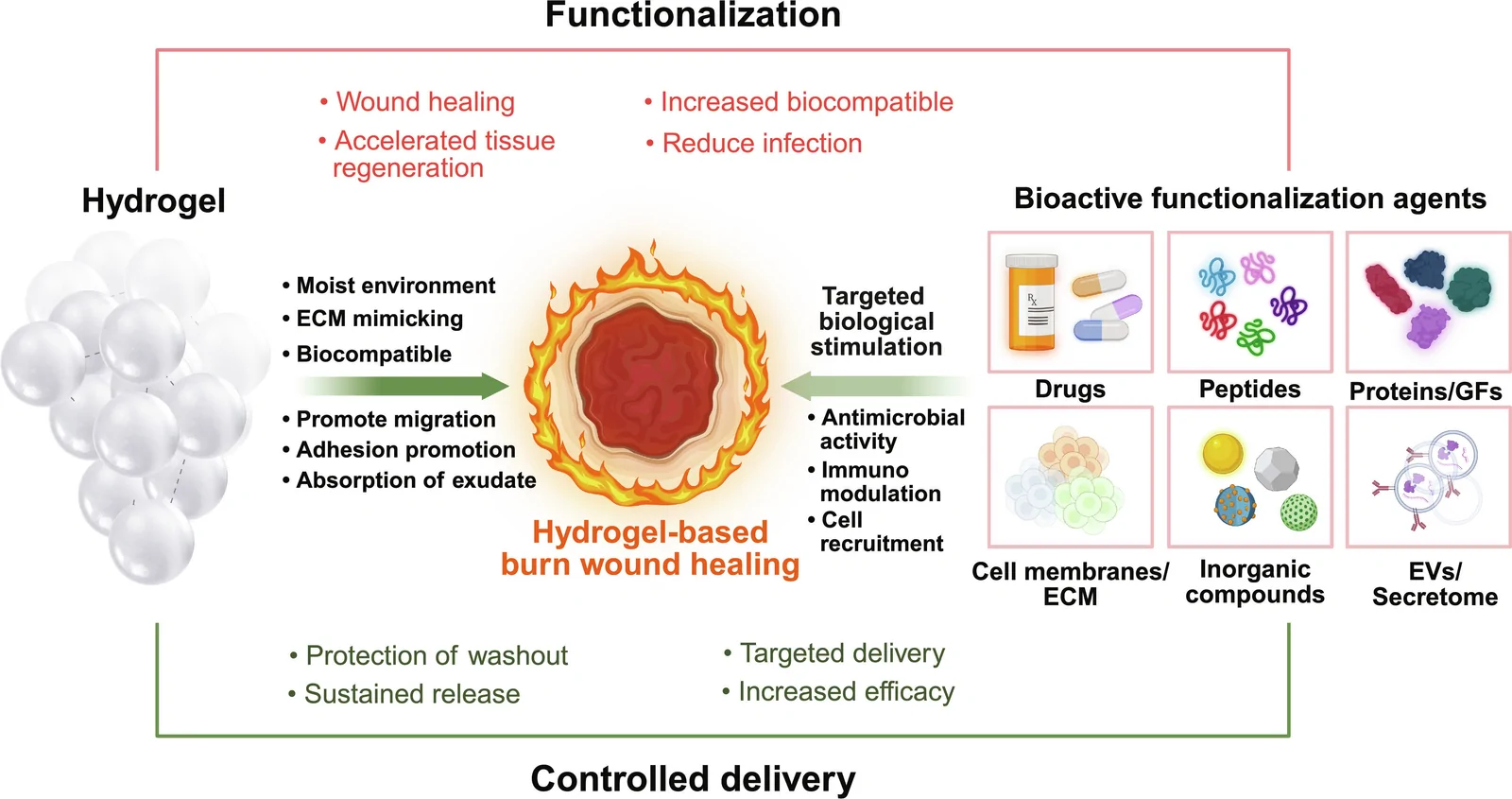
Schematic illustration of hydrogel-based wound-healing platforms. Hydrogels provide a moist, biocompatible, and ECM-mimicking microenvironment that supports cell adhesion, migration, and tissue regeneration while absorbing excess wound exudate. Functionalization of hydrogels with bioactive agents, including peptides, proteins, growth factors, antibiotics, nanoparticles, extracellular vesicles (EVs), and cell-derived components, enables targeted biological stimulation such as antibacterial activity, angiogenesis, and tissue repair. The hydrogel matrix protects these therapeutic agents from rapid washout and enables sustained, localized delivery, thereby enhancing therapeutic efficacy and accelerating wound healing. The illustration was created using BioRender.com.

### Peptide-based functionalization

Peptide-based functionalization has emerged as a highly promising strategy to enhance the bioactivity of hydrogels for burn wound healing. Peptides offer advantages over full-length proteins, including low immunogenicity, tunable sequences, chemical stability, and ease of synthesis. Peptide-functionalized hydrogels primarily target the inflammatory and proliferative phases of burn wound healing. During these stages, persistent bacterial contamination, ECM disruption, and impaired angiogenesis can significantly delay tissue repair. Peptide-functionalized hydrogels can be rationally designed with peptide-specific functional properties, including antimicrobial, cell-adhesive, and angiogenic properties, and can help reduce infection-associated inflammation while promoting keratinocyte migration, endothelial cell recruitment, and tissue regeneration [86].

Antibacterial peptide (AMP) incorporation using different methods is a highly demanding area of research, as burn wounds are vulnerable to bacterial infection, particularly by *Staphylococcus aureus* and *Pseudomonas aeruginosa*. AMP-functionalized hydrogels exhibit broad-spectrum antimicrobial activity via electrostatic interactions with bacterial membranes, membrane disruption, and intracellular interference. For example, Khan et al. [87] developed catechol-crosslinked antimicrobial peptide hydrogels by mussel-inspired chemistry between catechol groups and amine-rich polymers such as ε-poly-L-lysine. The prepared hydrogel formed a porous network with strong antimicrobial and antibiofilm activity against multidrug-resistant bacteria, including *Acinetobacter baumannii*. In vivo studies using infected burn wound models showed that the hydrogel significantly reduced bacterial burden while maintaining good cytocompatibility and tissue compatibility, suggesting its potential as an infection-resistant wound dressing biomaterial.

In another study, Zhou et al. [88] developed an injectable antimicrobial peptide hydrogel. The natural peptide Jelleine-1 is derived from royal jelly. The jelleine-1 hydrogel was formed by self-assembly of amphiphilic peptide sequences into nanofibers, followed by physical crosslinking to form a 3D hydrogel network. The resulting hydrogel possesses intrinsic antimicrobial activity, eliminating the need for additional antibiotics. In infected burn wound models, natural Jelleine-1 hydrogels demonstrated excellent biocompatibility, leveraging broad-spectrum antibacterial activity and significant promotion of wound healing, suggesting their potential as multifunctional biomaterials for treating drug-resistant wound infections.

ECM disruption in burn wounds challenges keratinocyte repair and fibroblast adhesion. To solve this challenge, hydrogel functionalization with cell-adhesive peptides is gaining attention. RGD-peptide integration in hydrogels enhances integrin-mediated cell attachment, activates focal adhesion kinase signaling, and promotes migration and proliferation [79,89]. In a study, an Arg-Gly-Asp-Ser (RGDS) peptide-modified nanofiber hydrogel was developed via pH-induced self-assembly of amphiphilic peptide molecules into β-sheet nanofibers, which subsequently organize into an interconnected hydrogel network. Integrating the RGDS bioactive epitope, these nanofibrous scaffolds mimic ECM architecture and interact with cell receptors to regulate cellular behavior. In vitro studies demonstrated enhanced proliferation of thermally damaged fibroblasts and endothelial cells cultured on RGDS-modified gels, indicating that the nanofiber scaffold provides both structural support and biochemical signaling conducive to tissue regeneration [90].

Insufficient vascularization in severe burns can delay healing. Researchers are developing angiogenic peptide-functionalized hydrogels. The prepared angiogenic peptide hydrogel can mimic VEGF domains to stimulate endothelial cell proliferation and capillary formation. For example, Zhang et al. [70] developed an injectable hydrogel with pro-angiogenic targeting cysteine-modified PR1P peptide (Fig. S1). The peptide was incorporated into the hyperbranched poly(ethylene glycol) diacrylate (polyPEGDA) and thiolated HA (HA-SH) hydrogel via Michael-type addition. The rapid in situ hydrogel formation properties enable effective coverage of irregular burn wounds. The PR1P peptide enhanced the recruitment and stabilization of endogenous VEGF, thereby improving the hydrogel’s angiogenic potential. In vitro studies demonstrated good cytocompatibility and significantly enhanced endothelial cell migration and tube formation in the presence of VEGF. In a murine deep second-degree burn model, the hydrogel significantly accelerated wound closure, increased collagen deposition, and promoted re-epithelialization. Mechanistically, the PR1P peptide enhanced neovascularization by activating the VEGF-Akt signaling pathway and reduced inflammatory responses by regulating macrophage polarization via the CaMKK-AMPK pathway. Overall, this injectable PR1P-functionalized hydrogel offers a promising strategy to enhance angiogenesis, suppress inflammation, and improve burn wound healing [70]. In addition to targeting specific epitopes, various peptides are incorporated into a hydrogel, which could exhibit promising regenerative properties by modulating angiogenic signaling pathways.

These hydrogels contain peptide sequences that bind angiogenic GFs such as VEGF and FGF-2, thereby enhancing vascularization during tissue repair. In burn injury models, these combined peptide hydrogels, in addition to promoting rapid re-epithelialization, increased granulation tissue formation, stimulated angiogenesis, and significantly down-regulated IL-6 expression, thereby improving wound progression [91,92].

Self-assembling peptides can form nanofibrous structures that closely resemble the native ECM architecture, advancing burn-healing research. Hydrogels can be prepared with self-assembling peptides either independently or as reinforcing networks within polymeric hydrogels. In a study, Loo et al. demonstrated a class of “smart” peptide hydrogels [93]. Ultrashort aliphatic peptides possess an inherent ability to self-assemble into helical nanofibers, forming biomimetic hydrogel scaffolds that are nonimmunogenic and noncytotoxic. These nanofibrous peptide hydrogels significantly accelerated wound healing in a rat partial-thickness burn model. A comparative study was conducted with the standard dressing Mepitel (silicone-coated polyamide net) and 2 peptide hydrogel formulations. The peptide hydrogels showed enhanced epithelial and dermal regeneration without exogenous GFs. After 14 d, the peptide hydrogels outperformed Mepitel, with 2 peptide hydrogels achieving wound closure rates of 86.2% and 92.9%, whereas wounds treated with Mepitel. As a promising alternative for burn treatment, their regenerative potential may be further enhanced by incorporating bioactive molecules, such as GFs and cytokines.

In a hydrogel system, integration of nanofibers and self-assembled peptides has proven to be multifunctional, improving the mechanical and biological properties of the hydrogel [94]. Wang et al. [95] revealed the mechanism of self-assembling short-peptide nanofiber gel scaffolds for the aesthetic repair of burns. RADA16-1 self-assembling peptide nanofiber hydrogels were prepared using an ultrasonic method. Ultrasonication fragmented RADA16-1 fibers into short segments that subsequently reassembled into a gel scaffold. The RADA16-1 hydrogel treatment significantly improved wound healing, hair follicle regeneration, hair growth length, and the expression of GFs (bFGF and EGF), indicating that short peptide nanofiber gel scaffolds effectively promote burn wound repair.

Despite the promising regenerative potential of peptide-functionalized hydrogels, several challenges remain for clinical translation. Burn wounds often contain elevated levels of proteolytic enzymes, including MMPs and neutrophil-derived proteases, which can accelerate peptide degradation and reduce therapeutic efficacy. In addition, rapid peptide diffusion from hydrogel matrices may limit long-term bioactivity at the wound site. To overcome these limitations, emerging approaches such as peptide cyclization, incorporation of D-amino acids, nanoparticle-assisted delivery, and stimuli-responsive release systems are being explored to enhance peptide stability, prolong retention, and improve therapeutic performance [96,97].

### Protein/GF-based functionalization

Protein functionalization of hydrogels represents a powerful strategy to enhance biological performance in burn wound management. Unlike small molecules or peptides, proteins such as GFs, cytokines, and enzymes exert complex biological signaling effects that regulate cell proliferation, angiogenesis, inflammation, and oxidative stress. However, protein instability, rapid degradation, and burst release remain major challenges that must be addressed through advanced delivery strategies [98]. GFs and cytokines are among the most widely incorporated biomolecules in hydrogel systems for wound and burn healing. The dysregulation of certain GFs and cytokines severely impairs wound-healing processes. GFs deficiency delays burn healing, which may be supplemented with a hydrogel dressing [29].

The use of GFs and protein primarily addresses deficiencies during the proliferative phase of burn wound healing compared with other nonsupplemented standard treatments [99,100]. In this proliferative phase, there are reduced levels of endogenous GFs, impaired angiogenesis, delayed re-epithelialization, and insufficient ECM deposition. However, hydrogel systems enable localized and sustained delivery of these GFs, improving therapeutic efficacy while reducing systemic exposure. A chitosan gel developed with epidermal growth factor (EGF) was applied to second-degree burn wounds in rats. This EGF gel showed faster epithelialization and healing compared with other treatments [101]. Strategies for hydrogel-assisted delivery of GFs were applied to burn wound healing, with a recombinant human granulocyte-macrophage colony-stimulating factor-gelatin hydrogel accelerating burn closure, offering promise for advancing burn dressings [102–104].

Liu et al. [105] developed a composite hydrogel by incorporating bFGF-loaded alginate microspheres (Ms) into a carboxymethyl chitosan (CMCS)–PVA network. When this composite hydrogel system was applied in a full-thickness burn model, it significantly accelerated wound healing. The treated wounds showed enhanced re-epithelialization and improved dermal regeneration. In addition, the hydrogel group exhibited the highest density of newly formed and mature blood vessels, indicating promoted angiogenesis during the healing process. Similarly, EGF was incorporated into amorphous polysaccharide hydrogels from chitosan/alginate, which reported rheological and water-retention properties of the hydrogel, a controlled release rate of EGF, promotion of cell proliferation, and acceleration of wound healing [106]. Because of the instability of the GF, it could be encapsulated in skin-permeable liposomes, which enhance the stability of the GFs in addition to their penetration efficiency. For example, bFGF was encapsulated in a liposome loaded into hydrogels, demonstrating stability and sustained release. In the deep second-degree scald mouse model, most encapsulated bFGF penetrated the dermis and facilitated healing, with hair regrowth [107]. Additionally, it was reported that bFGF released from hydrogels can modulate distinct inflammatory pathways such as the extracellular signal-regulated kinase (ERK) and tropomyosin receptor kinases (TRK) pathway, thereby attenuating inflammation in burn wounds [108].

Mai et al. [109] developed a smart, bFGF-containing hydrogel-based nanodelivery system. The photosensitizer sinoporphyrin sodium and PLGA-bFGF nanoparticles were embedded within a network of CMCS and sodium alginate hydrogel for sustained delivery (Fig. S2A). The PLGA-bFGF nanoparticles were first synthesized using a double-emulsion solvent-evaporation method and then incorporated into the CMCS/alginate hydrogel via EDC/NHS-mediated crosslinking. The prepared hybrid hydrogel supports sustained release and controlled delivery of both the photosensitizer and the GF. The photodynamic antimicrobial chemotherapy under mild photoirradiation (30 J/cm^2^, 5 min) with the hydrogel demonstrated the generation of ROS, resulting in excellent antibacterial and anti-biofilm activities against multidrug-resistant *S. aureus* (Fig. S2B and C). Moreover, both in vitro and in vivo results indicated that sustained release of bFGF from the hydrogel network structure promoted angiogenesis, collagen deposition, and re-epithelialization, thereby resulting in faster wound closure and improved healing outcomes (Fig. S2D).

In a study, Wang et al. [110] developed an in situ-forming hydrogel that targets anti-inflammatory and pro-angiogenic up-regulation during burn healing. The Resveratrol (Res) and VEGF plasmids were integrated into a hydrogel composed of chemically modified HA, Dex, and β-cyclodextrin. The final combined hydrogel accelerated rat splinted excisional burn healing, particularly through high-dose VEGF secretion from the hydrogel, which reduced the inflammatory microenvironment by down-regulating IL-1β and TNF-α gene expression and promoted microvascular formation by increasing intrinsic VEGF expression.

Although GF (proteins)-functionalized hydrogels have demonstrated significant therapeutic benefits in preclinical burn models, several challenges continue to hinder their clinical translation. These biomolecules are inherently unstable and susceptible to enzymatic degradation, denaturation, and loss of bioactivity during formulation and storage. Furthermore, uncontrolled burst release from hydrogel matrices may result in short-term supraphysiological concentrations followed by insufficient long-term exposure, thereby reducing therapeutic effectiveness and increasing treatment costs [97,111,112]. Advanced strategies, including nanoparticle-mediated encapsulation, affinity-based GF binding, multilayer delivery systems, and stimuli-responsive hydrogels, have been developed to improve GF stability and achieve sustained, localized release. Future research should focus on optimizing these delivery platforms while balancing therapeutic efficacy, manufacturing complexity, and regulatory requirements.

### ECM-based functionalization

ECM-based hydrogel systems are among the most biologically investigated strategies in biomaterial design for tissue regeneration [113]. Unlike specific biomolecule functionalization approaches, ECM-based systems provide an inclusive microenvironment composed of various proteins and bound signaling molecules that collectively regulate cellular activity [114–116]. By recapitulating native tissue architecture and biochemical composition, ECM-functionalized hydrogels actively guide regenerative remodeling rather than merely provide passive wound coverage. ECM-based materials are showing promise as a potential therapeutic strategy for burn repair [113]. ECM is either used directly to prepare a hydrogel or in combination with a polymeric hydrogel. Song et al. [113] review in detail the current ECM-hydrogel technology and its application in burn wound healing.

### Cell/cell-derived material-based biofunctionalization

Cells and cell-derived materials represent a fundamental shift from passive biomaterial scaffolding toward living or biologically active systems that dynamically respond to the burn wound microenvironment. Cell-based and cell-derived material-functionalized hydrogels primarily target the inflammatory, proliferative, and remodeling phases of burn wound healing. Cell integration exclusively provides all necessary biomolecules to the hydrogel and actively participates in tissue regeneration through paracrine signaling. Moreover, cell-derived material strategies, such as cECM, EVs, and sacrotomes, serve as biologically active cargo to provide specific functions where needed. These approaches aim to recapitulate the regenerative niche and overcome limitations associated with rapid protein degradation or single-factor delivery [117,118].

#### Cell-loaded hydrogels

Significant progress has been made over the last few decades in stem cell-loaded hydrogel treatments for wound healing, due to their ability to provide structural support while maintaining cell viability and function. Mesenchymal stem cells (MSCs), adipose-derived stem cells (ADSCs), bone marrow-derived MSCs, and induced pluripotent stem cell-derived progenitors have been widely investigated [47,119]. Direct cell therapy at the wound site faces numerous challenges, such as poor engraftment and rapid cell washout. Cell integration within hydrogels not only mitigates those challenges but also increases cell survival by shielding transplanted cells from mechanical stress, oxidative damage, and inflammatory mediators present in burn wounds. On the other hand, integrated cells can show their full potential and form biocompatible hydrogels [120,121].

In skin wound-healing processes, keratinocytes play a major protective role, which has inspired their use in cell therapy for burn treatment. Incorporation of human epidermal keratinocytes and human dermal fibroblasts in a hydrogel system resulted in significant improvement of burn wound healing compared to bare hydrogel. This combined hydrogel system increased collagen deposition and healing of full-thickness skin wounds in the sheep model [122,123].

As a promising advancement in burn treatments, stem cell incorporation into hydrogels is being investigated. Benefiting from MSCs’ ability to differentiate into various cell types, the MSC-laden hydrogel system can potentially promote skin tissue regeneration. For example, MSCs combined with a polysaccharide hydrogel, applied in a rat model of corneal alkali burns, promoted wound repair by up-regulating anti-inflammatory cytokines and the angiogenesis marker, and down-regulating inflammatory cytokines [124]. Similarly, MSCs were seeded on a biodegradable hydrogel scaffold prepared with an unsaturated arginine-based poly(ester amide) and a chitosan derivative, and applied in a mouse third-degree burn model. The results demonstrated that cell-laden hydrogel scaffolds enhance wound closure by faster epithelialization, granulation tissue formation, and vascularization in the burn site [125]. In a study, Rasouli et al. [126] prepared a hydrogel of carboxylated cellulose enriched with the KTTKS (Lys-Thr-Thr-Lys-Ser) peptide derived from the proteolytic hydrolysis of type I collagen, before MSCs seeding. The combined formulation showed significantly reduced inflammation, increased collagen deposition and angiogenesis, and improved burn wound closure. In a study, Mirshekar et al. [127] loaded a combination of bone marrow and adipose MSCs into a collagen-fibrin hydrogel. The results represent superior wound healing in the burn model caused by *P. aeruginosa*, compared to individual cell loading.

Several studies report that ADSCs loaded into different hydrogel formulations significantly improve burn healing [128–130]. Moreover, hydrogels loaded with ADSCs proved effective in regulating key molecules involved in burn repair. For example, ADSCs loaded with HA hydrogel prominently modulate IL-1β, transforming growth factor-β1 (TGF-β1), and bFGF when applied in rat burn dressing [131]. Dong et al. [79] developed an enhanced approach to deliver ADSCs for the treatment of burn wounds, using an in situ-formed hydrogel system comprising a commercially available thiol-functionalized HA (HA-SH) and a short RGD peptide (Fig. S3A). The results demonstrated that loaded cells in the hydrogel survived in the harmful wound environment, with significant retention capacity (Fig. S3B), enhanced neovascularization, accelerated wound closure, and reduced scar formation, all in a significantly shorter time than with bare hydrogels (Fig. S3C and D). Furthermore, researchers demonstrated various formulations and functionalizations of hydrogel systems with ADSCs, such as an injectable, self-healing hydrogel prepared via a Schiff-base reaction (Fig. S3E) [132] and an enzyme-crosslinked gelatin hydrogel (Fig. S3F) [133], which facilitated burn repair.

Codelivery of cells and other target agents is one of the improvements in producing a multifunctional hydrogel system. For example, Banerjee et al. [134] reported coloading of antibacterial silver sulfadiazine and ADSCs in a fibrin hydrogel. The dual effect of the hydrogel significantly reduced bacterial colonization and promoted neovascularization, with improved matrix remodeling. Further advances in codelivery were reported by Wu et al. [135]. ADSCs and nitric oxide (NO) were incorporated into 3D bioprinted hydrogel scaffolds. These systems showed increased neovascularization via the VEGF signaling pathway during wound healing in a mouse model.

#### Exosome-loaded hydrogels

Exosomes, nano-sized EVs secreted by stem and progenitor cells, have emerged as a cell-free alternative to whole-cell therapy. They contain bioactive cargo, including microRNAs, proteins, lipids, and signaling molecules that modulate angiogenesis, inflammation, and tissue remodeling [136–138]. Moreover, exosomes can deliver target-specific biomolecules to the burn site, which accelerates burn wound healing [139,140]. Wang et al. [118] recently reviewed in detail the application and roles of EVs in burn injury.

Hydrogel incorporation addresses the rapid clearance and short half-life of free exosomes by enabling sustained, localized release. In a study, an innovative exosome-loaded, sprayable, thermosensitive hydrogel was developed [141]. The exosome-functionalized hydrogels have demonstrated sustained exosome release and enhanced antibacterial activity, promoting granulation tissue formation and collagen deposition, and inducing hair follicle regeneration in a deep partial-thickness burn model. However, EVs are currently attracting attention across different areas of regenerative medicine; very few have been reported in hydrogel systems for burn repair.

#### Secretome delivery systems

The cell secretome encompasses soluble GFs, cytokines, chemokines, and EVs, collectively responsible for most regenerative effects [142,143]. Secretome-loaded hydrogels provide a cell-free strategy that avoids viability issues while maintaining bioactivity. Controlled-release systems have been developed to protect labile secreted factors from degradation and to enable sustained delivery. Compared to isolated GF incorporation, whole secretome delivery offers synergistic signaling and improved modulation of angiogenesis and inflammation [144–147].

The secretome was also investigated in wound and third-degree burn healing, reporting very promising results in wound repair by reducing inflammation, improving collagen and blood vessel formation, and reducing fibrotic complications [148,149]. The UCMSC-derived secretome was used to treat a 34-year-old male mining company worker after severe burn injury to his hand. Following treatment, the keloid’s texture, flexibility, and hand mobility significantly improved [150]. Furthermore, advances in secretomimetic therapy were combined with a hydrogel for burn treatments. Zhou et al. [151] developed a thermosensitive hydrogel comprising human umbilical cord MSC-conditioned medium/chitosan/collagen/β-glycerophosphate. The initially prepared materials can be stored at 4 °C in liquid form, while gel formation occurs upon application to burned mice at physiological temperature. The resulting hydrogel accelerated the formation of superior, well-vascularized granulation tissue and protected against the formation of fibrotic and hypertrophic scar tissue at the burn site. In a study, Kuncorojakti et al. [152] investigated a hydroxypropyl methylcellulose hydrogel incorporating adipose MSCs and compared it with a commercial hydrogel. The result indicated that it outperformed the commercial hydrogel in accelerating the reduction of the residual burn area and in promoting fibroblast proliferation and collagen density. However, compositional variability across cell sources, culture conditions, and donors poses challenges to reproducibility; further investigation is needed for clinical translation.

### Organic compound-based functionalization

Organic compounds, such as natural polyphenols, antioxidants, drugs, and antibiotics, offer a versatile, translationally attractive approach for wound treatment. Natural polyphenols, such as curcumin, have antibacterial activity [153], inhibit NF-κB signaling [82], and reduce pro-inflammatory cytokine production [83], thereby supporting wound repair [154]. Quercetin modulates ROS-mediated inflammatory signals [155], while resveratrol helps improve neovascularization and mitochondrial function in injured tissues [156]. However, the single application of these molecules is limited by poor water solubility and low bioavailability. Functionalization of hydrogels with such compounds at low doses could enhance solubility, protect against rapid degradation, and increase the hydrogel’s biological activity [154].

For example, Zhu et al. [157] prepared a self-healable chitosan hydrogel and incorporated the colistin lipopeptide as an antibiotic. The result demonstrated that the colistin-loaded hydrogel performed better than native colistin against both colistin-sensitive and colistin-resistant *P. aeruginosa* strains in the burn model. Additionally, the hydrogel system increased product degradation and sustained release, which is believed to enhance the formulation’s potential for localized burn healing. In another study, moxifloxacin loaded into a chitosan-based hydrogel [158], azithromycin encapsulated in silk sericin/PVA hydrogels by genipin crosslinking [159], and minocycline or gentamicin loaded into agarose hydrogel [160] were successfully used to treat biofilm-mediated infections during burn treatment. Similarly, different types of drugs, such as vancomycin, gentamicin, and minocycline, were loaded at high concentrations in alginate hydrogel. The hydrogel system was tested in a platform wound device (PWD) for burn treatment. This PWD and hydrogel technology offers a safe, controlled release of high-concentration drugs to treat burn infections [161] successfully. In a study, Li et al. [162] prepared a hydrogel by directly incorporating the antibacterial drug rhein into the backbone of aramid nanofibers. The hydrogel system maintains continuous drug release upon degradation, and the results suggest its application in *S. aureus*-associated burn wound infections.

From the literature, we found that researchers are extensively focusing on curcumin for various tissue-healing applications. However, very few reports have used curcumin in a hydrogel system for burn treatment. In a study, Gong et al. [163] reported a self-healing hydrogel system comprising an ε-poly-l-lysine (ε-PLL) polymer and poly(γ-glutamic acid) (γ-PGA), and a curcumin-loaded magnesium polyphenol network (Fig. S4A to D). The hydrogel system demonstrated strong hydrophilic and water-absorbing properties, facilitating rapid absorption of wound exudate by the Cur-Mg@PP powder and its conversion into a moist, viscous hydrogel at the injury site. This hydrogel system enables the controlled release of therapeutic agents, including magnesium ions (Mg^2+^) and curcumin (Cur). The released Mg^2+^ and Cur exhibit antibacterial activity against different bacterial strains (Fig. S4E to H). Additionally, the developed hydrogel system demonstrated multiple beneficial effects, including anti-inflammatory, pro-angiogenic, and tissue-regenerative activities, thereby accelerating burn wound healing.

In addition to microbial-targeted hydrogel formulations, several studies reported drug-loaded hydrogels designed for target-mediated burn healing. For example, a hydrogel was prepared by crosslinking of modified Dex and PEG diacrylate [164]. The prepared hydrogels have been shown to promote angiogenesis and dermal regeneration in deep burn wounds by facilitating endothelial cell infiltration and neovascularization. These scaffolds provide a supportive microenvironment for cell migration and tissue remodeling, thereby promoting improved vascularization, the formation of mature epidermal structures, and the regeneration of hair follicles.

More advanced systems include in situ-forming hydrogels for localized drug delivery or nanoparticle-hydrogel hybrid platforms for controlled, stimuli-responsive drug release. For instance, pH-sensitive silk vancomycin-loaded fibroin/alginate nanoparticles embedded in PNIPAM hydrogels, along with EGF [165]. In this dual-drug delivery system, nanoparticles enable pH-responsive antibiotic release in the alkaline microenvironment of infected wounds, while the hydrogel provides sustained EGF delivery to promote wound repair. This combined hydrogel system enhances antibacterial activity, fibroblast proliferation, re-epithelialization, and angiogenesis and has proven to be a promising candidate for severe burn treatments.

In another study, metformin hydrochloride was loaded into in situ-forming hydrogels composed of gelling agents such as Poloxamer 407, Carbopol, and sodium carboxymethylcellulose [166]. The result demonstrated that the hydrogel enables controlled transdermal drug release directly to the burn wound site, reducing systemic side effects while enhancing anti-inflammatory responses and tissue repair. In vivo studies demonstrated a significant reduction in burn wound size and accelerated healing over approximately 4 weeks of treatment.

### Nanoparticles-based functionalization

Nanoparticle-incorporated hydrogels combine the conformable properties of polymeric dressings with the functional advantages of nanoscale materials, offering a promising strategy for burn wound management [167]. Nanomaterial-functionalized hydrogels address multiple stages of burn wound healing, particularly inflammation and proliferation. Embedding nanoparticles (e.g., magnesium, silver, zinc oxide, and gold) enhances antimicrobial action, enables sustained and targeted delivery of therapeutic agents, and can improve mechanical strength, swelling behavior, and adhesive performance [168,169]. These composite systems can modulate inflammation, promote angiogenesis and re-epithelialization, and reduce dressing changes by providing prolonged activity at the wound site. Careful selection of nanoparticle type, concentration, and hydrogel matrix is critical to balance efficacy with biocompatibility and minimize cytotoxicity, making nanoparticle-laden hydrogels a versatile platform for accelerating burn healing [170,171].

Self-healing hydrogels were developed by forming crosslinks between polymer chains via ligand-metal coordination. By selecting different ligands and metal ions, e.g., copper (Cu^2+^), silver (Ag), and calcium (Ca^2+^), these gels can be tuned to exhibit a variety of mechanical properties suitable for diverse biomedical self-repair requirements [170,172]. For example, Kim et al. developed an injectable hydrogel with methylcellulose hydrogel containing silver oxide nanoparticles via a simple one-pot synthesis method. The resulting hydrogel exhibited controlled release of Ag^+^ ions, strong, broad-spectrum antimicrobial activity against various bacterial strains, and significantly improved burn wound healing in rat models.

In a study, Chen et al. [173] AgNPs embedded in a quaternized chitosan network to develop an antibacterial hydrogel for burn treatment. The resulting hydrogel system outperformed commercial Ag dressings in terms of antibacterial activity and burn wound healing. Severe burns disrupt the epithelium and sustain inflammation, delaying re-epithelialization and increasing the risk of infection. To address this, Chen et al. [174] further advanced burn dressings by developing a multifunctional smart hydrogel (Ag-IMP-U@Cur). Uridine is incorporated into the network via reversible boronate ester linkages with phenylboronic acid, which confer pH responsiveness and promote re-epithelialization. An Ag-IMP complex reinforces the scaffold, while Cur-loaded ZIF-8 (Cur-ZIF-8) provides controlled release with antibacterial and anti-inflammatory effects. Unlike typical hydrogels with limited functions, Ag-IMP-U@Cur combines uridine-driven tissue repair, Ag-IMP structural/antimicrobial roles, and Cur-ZIF-8 therapeutic delivery. In a rat full-thickness burn model, wounds treated with the hydrogel healed faster than controls. Moreover, depending on the inorganic compound’s activity in the biological system, a functionalized hydrogel can be selectively developed for immunomodulatory-mediated burn healing. For example, Zhao et al. prepared a multifunctional hydrogel (GTMG) by incorporating polydopamine-modified graphene oxide (GA), tannic acid (TA), and Mg^2+^ ions into a gelatin methacrylate (GelMA) matrix through a simple photocrosslinking method [175]. The hydrogel system rapidly forms a 3D porous hydrogel network under visible-light irradiation (405 nm) and shows good injectability, adhesion, and shape adaptability. The composite hydrogel demonstrated excellent cytocompatibility, antioxidant, antibacterial, and hemostatic properties, while effectively regulating the immune microenvironment by promoting macrophage polarization from the pro-inflammatory M1 phenotype to the regenerative M2 phenotype. The hydrogel showed accelerated tissue regeneration, improved vascularization, and shortened the overall wound-healing time when applied in rat burn models, indicating its strong potential as a multifunctional dressing for burn treatment.

### Stimuli-responsive assisted functionalized hydrogel systems

Apart from bioactive material-based functionalization of hydrogels, stimuli-responsive hydrogel systems represent an emerging smart generation of wound dressings capable of dynamically interacting with the burn wound microenvironment. Unlike conventional functionalization systems, these smart hydrogels respond to local physicochemical environments, including changes in electrical, light, temperature, and pH, as well as ROS, enabling on-demand release of bioactive materials. In this system, the activity of the functionalization material relies on the stimulation of the host microenvironment, with the hydrogel formulated to respond to the target stimulus. Given the highly dynamic and pathological nature of burn wounds, including acidosis, oxidative stress, bacterial colonization, and elevated protease levels, stimuli-responsive materials enable control of the bioactive material’s activity within the hydrogel system [176–178]. These responsive hydrogels are generally prepared by combining functional monomers and crosslinkers via polymerization and crosslinking, followed by direct incorporation or crosslinking of functionalization material. For example, a chitosan hydrogel was prepared by crosslinking with β-glycerolphosphate disodium salt pentahydrate, which forms a hydrogel at 37 °C from a gel state at 4 °C. This hydrogel is potentially applicable in body-temperature environments and is excellent for the management of burn wounds infected with extensively drug-resistant bacteria [179].

Moreover, thermoresponsive hydrogel is smartly designed with multifunctional purposes. Xie et al. [180] prepared hydrogel poly(ethylene glycol)-block-poly(L-methionine) (EG45Met25), which not only forms a stable hydrogel network at physiological temperatures but also confers intrinsic antioxidant activity through ROS-mediated oxidation to sulfoxide/sulfone. The antioxidant properties of hydrogel synergistically promote chronic wound repair via down-regulating macrophage-mediated inflammatory cytokines. Because of the formation of hydrogel at a host’s normal temperature, in burn therapy, a thermoresponsive system assists in retaining functionalized bioactive agents and reduces leakage from irregular wound geometries. However, unexpected phase transitions may cause burst release of incorporated materials. Furthermore, long-term biocompatibility and degradation behavior of certain synthetic thermoresponsive polymers require careful evaluation.

Like thermosensitive systems, pH-responsive hydrogels exploit ionizable functional groups and acid-labile linkages that undergo swelling, degradation, or bond cleavage at the host pH. Such systems enable targeted bioactive release in infected or inflamed tissues, depending on physiological conditions. As burn and infected wounds often exhibit acidic microenvironments (pH 5.5 to 6.5), the resulting hydrogel not only is effective against bacterial infection and burn healing but also helps prevent the overuse of medication after healing [17].

Unlike the host microenvironment, the electroactive, photothermally responsive hydrogel is designed to release bioactive molecules in response to external stimuli. Electroactive and photothermal-responsive platforms have emerged as highly promising tools for multimodal therapy [181]. Severe burn injuries destroy the skin’s endogenous electrical fields, which are normally vital for directing cellular migration during repair [182]. Electroactive hydrogels act as mechanoelectrical transducers, converting mechanical stimuli into biologically relevant electrical signals that can regulate a broad range of biological processes. Electroactive materials could be crosslinked via light irradiation, facilitating on-demand release of a bioactive agent [183,184].

Li et al. are advancing a conductive organohydrogel system incorporating protein and enzyme. The hydrogel combined a physically crosslinked, rigid network with a chemically crosslinked, bioactive soft network, resulting in excellent mechanical strength, conductivity, and antifreeze properties. This hydrogel, when applied in a mouse model of fireworks burns, rapidly cooled burn wounds and reduced inflammation and tissue damage. Additionally, its conductive and flexible nature enabled real-time monitoring of joint movements associated with wounds, highlighting its potential as an intelligent wound-healing and wearable sensing platform [185].

Furthermore, to address the severe risk of multidrug-resistant infections and sepsis, which are common in extensive burns, the functionalization of hydrogel dressing with photothermal agents, such as carbon nanotubes, carbon dots or polymer dots, MXene, and metal nanoparticles, enables spatiotemporally controlled therapeutic interventions [186–188]. This strategy can execute a highly localized antimicrobial mechanism, wherein the targeted near-infrared (NIR) irradiation induces localized hyperthermia that physically disrupts established biofilms and bacterial cell membranes, all while sparing the surrounding nascent tissue from thermal damage. Stimuli-responsive functionalized hydrogels represent a transformative approach for adaptive burn management. By aligning therapeutic release with pathological signals, these systems enhance efficacy while minimizing side effects and drug resistance. However, challenges remain in balancing responsiveness with mechanical stability, achieving reproducible manufacturing, and ensuring safety under variable wound conditions [189]. Electric output, controlled release, protection of bioactive materials from damage, and mechanical stability remain challenges to be considered in the future for the formulation of a stimuli-responsive hydrogel to achieve precision-guided burn regeneration [190]. To address the limitations of the external stimulation system, incorporating a humidity-responsive moisture-electric generator into the hydrogel system could be effective for autonomously delivering bioactive molecules [191].

### Other sources of hydrogel functionalization materials

In addition to conventional sources of biofunctional materials for preparing and enhancing hydrogel efficacy in burn treatments, nature-inspired or plant extract sources have been investigated for their potential**.** For example, Zohdi et al. [192] developed a Malaysian sea cucumber-based hydrogel (Gamat Hydrogel) combining polyvinyl pyrrolidone and PEG via electron beam crosslinking. The Gamat Hydrogel was applied in a rat burn model for 28 d, and histological and molecular evaluations were conducted. The Gamat Hydrogel enhanced burn wound repair by modulating tissue regeneration and down-regulating pro-inflammatory cytokines, including IL-1α, IL-1β, and IL-6. The results demonstrated that the Gamat Hydrogel outperforms the control hydrogel and the commercial OpSite film dressing, as shown in the study. Given the matrix structure and structural support, many available plant extracts, oils, or ointments can be used in a hydrogel system. Research has found that these approaches can increase the effectiveness of hydrogel and biological components [193]. Wang et al. [194] crosslinked Eucalyptus essential oil, ginger essential oil, and cumin essential oil in a polysaccharide-based hydrogel. They found it effective in antibacterial-mediated burn healing.

### Commercially available hydrogel products

Several hydrogel-based products are commercially available for burn wound management (Table 2), each designed to maintain a moist healing environment while providing cooling, pain relief, and wound protection. Water-Jel, developed by Water-Jel Technologies (Carlstadt, NJ, USA), the original inventor and manufacturer of gel-soaked burn dressing technology since 1979, consists of a bacteriostatic water-soluble hydrogel matrix with a thick carrier system that provides nonadherent coverage and effective cooling during emergency burn care. Burn Shield is a water-based hydrogel dressing infused with *Melaleuca alternifolia* (tea tree oil) that offers immediate cooling and hydration to burn injuries. Amorphous hydrogel formulations such as Purilon Gel, composed primarily of purified water, sodium carboxymethylcellulose, and calcium alginate, promote hydration and autolytic debridement, whereas Intrasite Gel contains partially hydrated carboxymethylcellulose, propylene glycol, and purified water to support moisture balance and wound healing. Similarly, Solosite Gel utilizes a highly hydrating water-swellable polymer matrix with preservatives to limit microbial contamination. Hydrogel sheet dressings such as Vigilon, a crosslinked polyethylene oxide copolymer matrix suspended in water, provide a flexible, transparent, and nonadherent wound covering. Likewise, Neoheal is a radiation-crosslinked hydrophilic polymer network containing more than 90% water, offering excellent biocompatibility, elasticity, transparency, and sustained wound hydration. Together, these products demonstrate the broad clinical utility of hydrogel technologies in burn care, ranging from emergency first aid and pain management to moisture retention, autolytic debridement, and support for the wound-healing process.

**Table 2.** Commercially available hydrogel products used in burn wound management and their key characteristics

Product name	Company name	Type	Features
Water-Jel	Jackson Allison Medical & Surgical Ltd	Sheet dressing/Bottled gel	- Bacteriostatic water-soluble hydrogel matrix with a thick carrier system - Nonadherent and easy to apply
Burn Shield	Levtrade International (PTY) LTD	Sheet/Dressing pad	- Water-based hydrogel infused with natural *Melaleuca alternifolia* (tea tree oil) - Provides immediate cooling and a moist wound environment
Purilon Gel	Coloplast	Amorphous gel	- High-viscosity hydrogel composed primarily of purified water, sodium carboxymethylcellulose, and calcium alginate - Promotes moisture donation
Intrasite Gel	Smith & Nephew	Amorphous gel	- Partially hydrated carboxymethylcellulose polymer containing propylene glycol and purified water - Facilitates moisture balance
Solosite Gel	Smith & Nephew	Amorphous gel	- Water-swellable polymer matrix with preservatives to limit microbial contamination - Highly hydrating formulation
Vigilon	C.R. Bard	Hydrogel sheet dressing	- Crosslinked polyethylene oxide (PEO) copolymer matrix suspended in water - Flexible, transparent, and nonadherent
Neoheal	KIKGEL	Hydrogel sheet dressing	- Radiation-crosslinked hydrophilic polymer network with >90% water content - Transparent, elastic, and biocompatible

## Mechanistic Basis of Hydrogel-Mediated Burn Repair: Biological and Molecular Perspectives

Functionalized hydrogels strengthen therapeutic effects in burn healing through multifaceted biological mechanisms that extend beyond the physicochemical properties of simple hydrogels. Advancing the hydrogel system by incorporating bioactive molecules, cells, organic/inorganic nanoparticles, or active components that actively engage in infection and inflammation control enhanced vascularization, epithelial regeneration, and ECM remodeling, and reduced scar formation. We summarize the mechanistic basis of hydrogel-mediated burn repair by functionalizing hydrogels in Fig. 3.

**Fig. 3.**
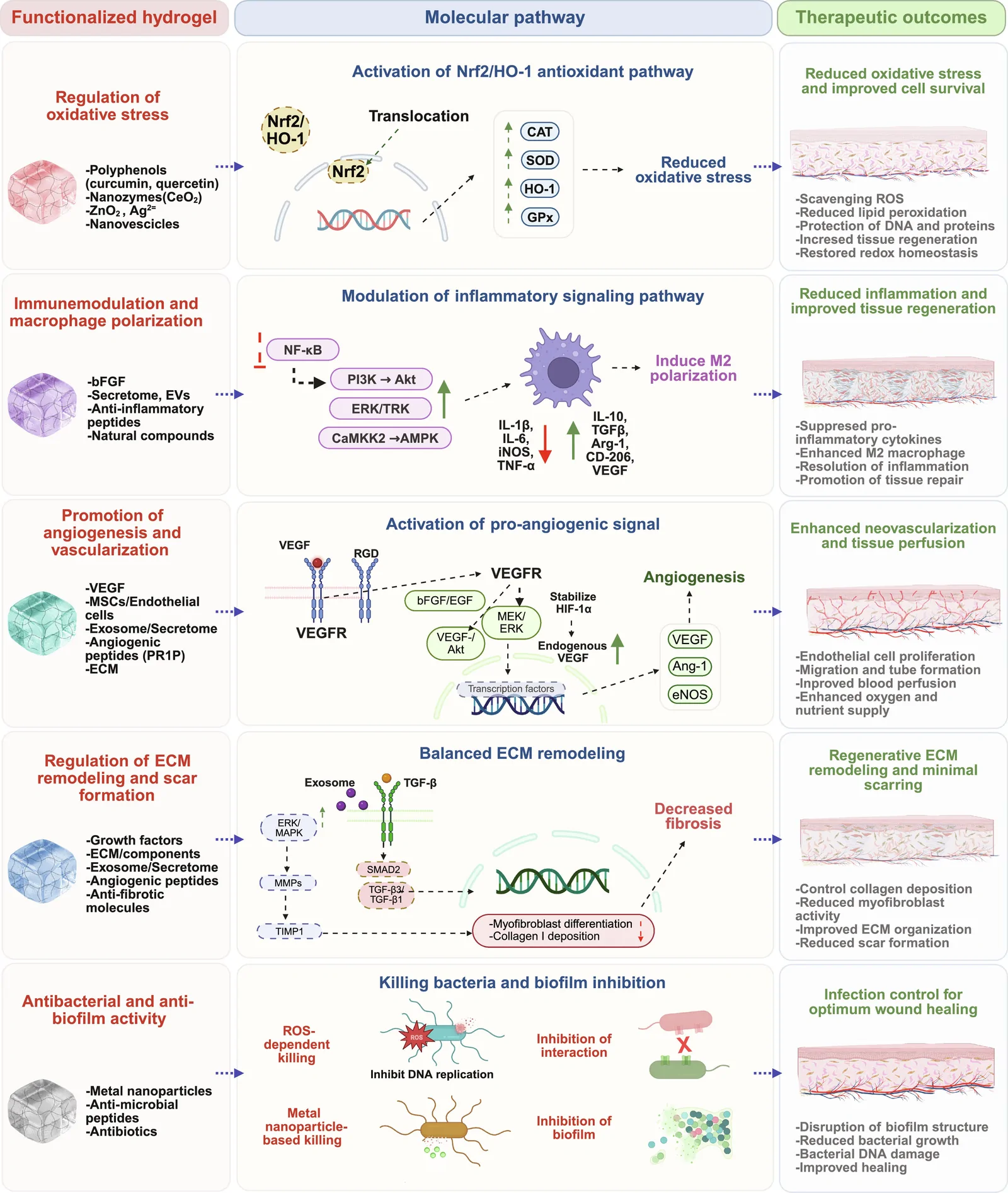
Schematic presentation of a summary of biological and molecular events enhanced by bioactive components in functionalized hydrogel systems during wound healing. The illustration was created using BioRender.com.

### Antibacterial and anti-biofilm by functionalized hydrogels

Burn wounds are highly susceptible to bacterial colonization and biofilm formation due to compromised barrier integrity, the presence of necrotic tissue, and immune dysregulation. Common burn-associated pathogens, including *P. aeruginosa*, *S. aureus*, and *A. baumannii*, can form structured biofilms composed of extracellular polymeric substances that protect bacterial communities from antibiotics, immune cell clearance, and environmental stresses [87,170].

Targeted antimicrobial therapy via a hydrogel system leverages multiple mechanisms, including sustained antibiotic release, nanoparticle-mediated membrane disruption, ROS generation, and antimicrobial peptide activity, to enhance burn healing [3,195]. The most common approach to address wound infection challenges is either to inhibit bacterial growth and biofilm formation or to use contact killing. Various drugs and AMPs are excellent candidates for functionalizing hydrogels to combat microbial infections, as inert hydrogels are insufficient. The physical properties of the hydrogel matrix act as a responsive material, with a changing microenvironment upon infection and, accordingly, the release of bioactive molecules [3,195,196]. Because of drug resistance, inorganic nanoparticles and naturally derived antioxidant agents are promising candidates for strengthening hydrogel dressings. Incorporating these compounds into the hydrogel effectively reduces bacterial infection by killing bacteria. The production of ROS by functionalized hydrogels disrupts bacterial membrane integrity and permeability, leading to leakage of intracellular components and impairment of essential metabolic processes. ROS production also induces bacterial DNA damage, strand breakage, structural disintegration, and the formation of stable oxidation products [170,197]. Additionally, they accelerated bacterial infection-mediated burn wound healing by macrophage polarization and stimulating angiogenesis. Therefore, optimizing release kinetics and concentration in the hydrogel system promises to balance bactericidal efficacy with cytocompatibility [17,175].

### Regulation of oxidative stress by functionalized hydrogels

Oxidative stress is a hallmark of burn wound pathology and plays a critical role in delaying tissue repair. Following thermal injury, activated neutrophils, macrophages, and damaged tissue cells generate excessive ROS, including superoxide anions, hydrogen peroxide, and hydroxyl radicals [197]. Functionalized hydrogels can effectively regulate oxidative stress by incorporating antioxidant compounds such as curcumin [163], quercetin [155], resveratrol [156], and chlorhexidine [198], as well as ROS-scavenging nanoparticles and enzyme-mimetic nanozymes [199]. These bioactive agents directly scavenge free radicals while simultaneously activating endogenous antioxidant defense mechanisms [170]. The programmed antioxidant hydrogel also creates a favorable microenvironment for tissue regeneration by modulating several molecular pathways. The synergistic ROS scavenging effect potentially suppresses inflammation via the MAPK/NF-κB pathway and the TICAM-1 pathway [200]. A key molecular pathway involved is the nuclear factor erythroid 2-related factor 2 (Nrf2)/heme oxygenase-1 (HO-1) signaling axis. Upon activation, Nrf2 translocates into the nucleus and promotes the expression of antioxidant enzymes, including superoxide dismutase, catalase, glutathione peroxidase, and HO-1. Consequently, ROS levels are reduced, cellular damage is minimized, and a favorable microenvironment for tissue regeneration is established. By modulating oxidative stress and enhancing cellular redox homeostasis, antioxidant-functionalized hydrogels accelerate burn wound healing [198,201].

### Immunomodulation and macrophage polarization

The inflammatory response plays a central role in burn wound healing; however, prolonged inflammation often leads to chronic tissue damage and fibrosis. Macrophages are key regulators of this process and exhibit remarkable phenotypic plasticity. Following burn injury, pro-inflammatory M1 macrophages dominate the wound site and secrete cytokines such as TNF-α, IL-1β, IL-6, inducible nitric oxide synthase, and ROS. While these mediators contribute to pathogen clearance, excessive or prolonged M1 activation delays healing and exacerbates tissue damage [31].

Alternatively, a key therapeutic target is the macrophage polarization transition. GFs, extracellular vesicles, stem cell-derived secretomes, bioactive peptides, and natural anti-inflammatory compounds are incorporated within hydrogel matrices as immunomodulatory cues. These compounds release from the hydrogel to induce macrophage polarization, which produces anti-inflammatory and regenerative factors, including IL-10, TGF-β, arginase-1, and VEGF, which mediate neovascularization during burn healing [202]. Mechanistically, hydrogel-mediated immunomodulation often involves inhibition of NF-κB activation [82], resulting in reduced expression of TNF-α, IL-1β, and IL-6 [167]. Simultaneously, activation of PI3K/Akt [70] and CaMKKβ-AMPK signaling pathways promotes M2 polarization and the resolution of inflammation. Extracellular vesicles and secretomes also contribute by regulating inflammatory gene expression through microRNA-mediated mechanisms. Through coordinated modulation of immune responses, functionalized hydrogels facilitate the transition from inflammation to tissue regeneration, thereby improving healing outcomes in burn wounds [108].

### Promotion of angiogenesis and tissue vascularization

Adequate vascularization is essential for successful burn wound healing because newly formed blood vessels provide oxygen, nutrients, immune cells, and GFs necessary for tissue regeneration. Severe burns frequently disrupt the local vascular network, creating hypoxic conditions that impair fibroblast function, collagen synthesis, and epithelial regeneration. Consequently, insufficient angiogenesis is a major barrier to effective healing, particularly in deep partial-thickness and full-thickness burns.

Functionalized hydrogels promote neovascularization by sustaining the delivery of pro-angiogenic biomolecules. The incorporation of the angiogenic peptide, such as PR1P, into a hydrogel system directly modulates the VEGF-Akt signaling pathway via endogenous VEGF recruitment and stabilization [70]. Hydrogels designed to induce expression of GFs bFGF and EGF lead to ERK pathways that stimulate endothelial cell proliferation, migration, and capillary tube formation [95,108,165]. Alternatively, bioactive molecules such as proteins or GFs can be directly incorporated to regulate different angiogenesis-mediated pathways [94,101]. Activation of endothelial nitric oxide synthase further enhances vascular permeability and blood vessel maturation. In addition, certain molecules, such as the integrin-binding RGD peptide incorporated into hydrogel systems, stabilize hypoxia-inducible factor-1α, thereby promoting endogenous VEGF expression under hypoxic conditions [203]. Stem cell-loaded hydrogels, extracellular vesicle-functionalized hydrogels, and ECM-derived biomaterials further enhance angiogenesis by providing multiple synergistic pro-vascular signals. Collectively, these mechanisms contribute to improved tissue perfusion, accelerated granulation tissue formation, and enhanced regenerative healing [135].

### Regulation of ECM remodeling and scar formation

Recovery of the epidermal barrier during burn healing is a crucial phase that depends on faster epithelialization mediated by keratinocytes. During normal healing, a temporary ECM is gradually replaced by a collagen-rich, organized tissue. However, severe burn injuries frequently disrupt this process through persistent inflammation, excessive protease activity, and aberrant fibroblast activation, resulting in pathological fibrosis and hypertrophic scar formation. Elevated levels of MMPs, particularly MMP-2 and MMP-9, degrade collagen, fibronectin, laminin, and GFs, thereby impairing tissue reconstruction. Simultaneously, excessive TGF-β signaling promotes differentiation of fibroblasts into contractile myofibroblasts, leading to excessive collagen deposition and scar contracture.

Functionalized hydrogels help restore balanced matrix remodeling through multiple mechanisms. ECM-derived biomaterials, collagen, gelatin, HA, and bioactive peptides provide structural and biochemical cues that support cell adhesion, migration, and matrix synthesis. Functionalized hydrogels can involve remodeling processes at the cellular level, starting with enhancing adhesion-mediated signaling pathways and progressing to the tissue matrix. Moreover, the functional hydrogel network is mimicked by the ECM architecture [79,89,90,93].

GF-loaded hydrogels stimulate fibroblast activity and collagen organization, while extracellular vesicles and stem cell-derived secretomes regulate fibroblast behavior through paracrine signaling. At the molecular level, these systems can activate the ERK/MAPK pathway in skin dermal fibroblasts, consequently modulating the MMP/TIMP balance, suppressing excessive TGF-β/SMAD2 signaling, and inhibiting persistent myofibroblast activation. Furthermore, enhanced angiogenesis and resolution of inflammation indirectly contribute to constructive matrix remodeling by restoring tissue homeostasis. By regulating ECM turnover and fibrotic signaling pathways, functionalized hydrogels promote regenerative healing while minimizing hypertrophic scar formation and tissue contracture [137,204].

## Challenges and Future Perspectives of Functionalized Hydrogels in Burn Therapy

Despite substantial progress in developing functionalized hydrogels for the treatment of burn injuries, several complex technological and translational barriers continue to impede their widespread clinical adoption. Their compelling bioactivity in preclinical settings underscores their strong therapeutic potential. Yet, successful clinical translation demands a careful, strategic balance of stability, controlled and predictable release, mechanical integrity, manufacturability at scale, sterilization compatibility, and economic feasibility. Resolving these challenges is fundamental to advancing these systems from promising experimental platforms into dependable, clinically deployable therapies that can redefine standards of care and deliver tangible benefits to patients.

### Translational limitations of preclinical burn models

The challenges regarding treatment of burn-related wound healing are also exacerbated by the lack of clinical trials and, more importantly, many terminated and incomplete studies (Table 3). The analysis showed that only a handful of studies are registered and completed on the official clinical trials website.

**Table 3.** Clinical trials with hydrogel dressing for burn-related wounds

Name of the study	Treatment	Measure description	Status	Comment	NCT Number
Partial thickness burns	Hydrogel (Trademark: Burnshield)	Time to complete re-epithelialization (days), assessed up to 21 d	Terminated	Stopped by the sponsor due to their change in product development priorities	NCT03190655
Burn injury	Prontosan Wound Gel X	Outcomes assessed over 29 d included graft neo-epithelialization, time to complete epithelialization, wound infection, and need for reoperation at the target wound site	Completed	PWX was effective, safe, and well-tolerated for cleansing and maintaining moisture in deep partial- and full-thickness burns requiring split-thickness skin grafting	NCT01534858 [245]
Trial of different wound dressings in split-skin grafted third-degree burns	Hydrogel	Percentage of epithelialized wound area over time (up to 1 year)	Unknown	Recruiting	NCT03674151
Burn injury	-Altrazeal flexible hydrogel nanoparticle -Aquacel AG, a typical carboxymethylcellulose dressing	Range of motion of affected joints during treatment (up to 2 weeks)	Terminated	Lack of enrollment	NCT01062191
Burn injury	Synepure wound cleanser and Catasyn advanced technology hydrogel	Proportion of wounds achieving ≥90% re-epithelialization (up to 21 d)	Terminated	Lack of enrollment	NCT05877638
Superficial partial-thickness burn study	Catasyn Advanced Technology Hydrogel and SynePure Wound Cleanser	Change from screening visit to visit 8, in healing of the superficial partial thickness burn wound (time frame: up to 21 d)	Terminated	Delayed recruitment	NCT04601532
Efficacy of MySkin patch for the healing of burn wounds	MySkin patch	Wound healing rate and reduction in pain intensity over 1 month	Unknown	Recruiting	NCT01499264
Effectiveness of KeraStat gel for improved cosmesis of partial-thickness burns	KeraStat Gel	Scar quality assessed using the patient and Observer Scar Assessment Scale at 1 year	Terminated	Low enrollment	NCT03564795
Allogeneic ADSCs and platelet-poor plasma fibrin hydrogel to treat patients with burn wounds (ADSCs-BWs)	Allogeneic ADSCs and platelet-poor plasma fibrin hydrogel	Degree of epithelialization and healing of grafted areas (up to 1 month)	Unknown	Recruiting	NCT03113747
A follow-up study to evaluate the safety of ALLO-ASC-DFU in ALLO-ASC-BI-101	ALLO-ASC-DFU (a hydrogel sheet containing allogenic adipose-derived mesenchymal stem cells)	Incidence of adverse events (safety and tolerability), monitored up to 24 months	Completed	Results not provided	NCT03183622
A follow-up study to evaluate the safety of ALLO-ASC-DFU in ALLO-ASC-BI-201	ALLO-ASC-DFU (a hydrogel sheet containing allogenic adipose-derived mesenchymal stem cells)	Incidence of adverse events (safety and tolerability), monitored up to 24 months	Completed	Results not provided	NCT03183648
Effectiveness comparison of skin micro-grafts vs. meshed split-thickness skin grafts	Skin micro graft	Percentage of wound epithelialization measured via imaging at day 14	Unknown	Recruiting	NCT02813213
Clinical registry collecting real-world evidence on wound care treatments	Various types of wound care treatments	Percentage reduction in wound area and incidence of treatment-emergent adverse events (up to 12 weeks)	Enrolling	Enrolling	NCT06328010

Although rodent burn models have substantially advanced the development of hydrogel-based therapies, important physiological differences exist between rodents and humans that may influence translational outcomes. Rodent skin is thinner, contains a dense panniculus carnosus muscle layer, and relies more heavily on wound contraction for closure. In contrast, human burn wounds predominantly heal through re-epithelialization and granulation tissue formation [205]. Furthermore, achieving consistent, reproducible burn depth remains challenging because burn severity can vary with temperature, contact time, pressure, and anatomical location. Differences in immune responses also exist, as rodents often exhibit inflammatory and regenerative responses that do not fully recapitulate human burn pathology. Consequently, therapeutic efficacy observed in small-animal models may overestimate clinical performance. While rodent models remain valuable for mechanistic investigations and initial therapeutic evaluation, large-animal models, particularly porcine burn models, more closely resemble human skin architecture, wound-healing kinetics, vascularization, and immune responses [205,206]. Therefore, future studies should emphasize standardized burn induction protocols and validation in clinically relevant large animal models to improve the translation of advanced hydrogel technologies into human burn care.

Regarding the issue, it can be assumed that, as burn injuries are critical, painful, and traumatic for the patients and their family members, recruiting patients for the study is very time-consuming and costly due to complications related to the research design. Medical practitioners and researchers need to collaborate across countries and accumulate and share data to understand better. More importantly, research collaborations between laboratories with advanced technologies need to engage low-income countries, where more patients can be recruited for clinical trials, which may yet be prevalent.

### Instability of functionalized bioactive molecules

Another critical barrier to advancing functionalized hydrogels for burn therapy is the instability of key bioactive molecules, including GFs, cytokines, enzymes, exosomes, and peptides, despite their essential roles in controlling inflammation, promoting cell proliferation and migration, driving angiogenesis, and enabling effective tissue remodeling in burn wounds. Bioactive molecules are highly sensitive to environmental stressors, including elevated temperature, pH fluctuations, shear forces during processing, and exposure to ROS or proteolytic enzymes. Such conditions may result in denaturation, oxidation, aggregation, or proteolytic cleavage, thereby reducing or eliminating their biological activity [97,112,207,208].

These physicochemical mismatches also critically hamper the sustained release of therapeutic agents or bioactive compounds [117,209]. The rapid initial diffusion of these substances can lead to excessively high local concentrations, potentially inducing cytotoxic effects and accelerating premature exhaustion of the therapeutic payload. Consequently, the development of delivery systems that closely mimic physiological release kinetics remains a critical priority in ongoing research. The simultaneous incorporation of multiple bioactive agents requires more care and the design of their release kinetics to ensure effective functioning. The challenge is to provide a more robust approach that protects and releases multiple agents at a time, with controlled release on demand. The delivery system and protection of the functionalization agent also depend on hydrogel formulation techniques, which introduce an additional layer of complexity. Numerous methods, especially those that rely on ultraviolet (UV) irradiation or free-radical polymerization, generate reactive species during network formation that can harm delicate biomolecules. These free radicals can oxidize amino acid residues or disrupt protein tertiary structure, while prolonged exposure to UV light may trigger photodegradation [210]. This leads to a key design compromise: achieving the intended hydrogel architecture and mechanical performance can compromise the functionality of embedded therapeutic molecules [211].

In contrast, milder strategies such as enzymatic crosslinking, ionic interactions, Schiff-base reactions, or visible-light-initiated approaches can reduce damage, though they may limit the ability to fine-tune gelation rates, mechanical properties, or degradation behavior. However, a tightly organized network often imposes significant limitations on key biological processes, including cellular infiltration, nutrient and oxygen diffusion, and regulated biodegradation, all of which are essential for effective tissue repair [212]. On the other hand, inorganic nanoparticles, which are more stable in the biological microenvironment, such as silver, zinc oxide, and cerium oxide, offer strong antimicrobial, antibiofilm, antioxidant, and immunomodulatory properties. However, concerns remain regarding long-term biocompatibility and dose-dependent cytotoxicity. For example, prolonged exposure to high concentrations of Ag^+^ ions can impair endothelial cell proliferation, reduce tube formation, increase oxidative stress, and inhibit collagen synthesis, thereby negatively affecting angiogenesis and tissue regeneration. Similar concerns have been reported for copper-based and ROS-generating antimicrobial systems. Therefore, antimicrobial components must be carefully optimized to maintain antibacterial activity while minimizing damage to host regenerative cells [170,212]. Hybrid approaches that integrate synthetic polymers for robust mechanical reinforcement with natural biomaterials for enhanced bioactivity represent a promising strategy. Nevertheless, achieving the ideal synergy in which mechanical integrity and biological functionality are both maximized without compromising either remains a complex, application-specific optimization challenge. However, integrating multiple therapeutic functions within a single platform also introduces significant biological and engineering challenges.

### Translational limitations to industrial scale

Expanding hydrogel fabrication from controlled laboratory conditions to large-scale production for clinical application presents considerable challenges. While small-scale preparation enables precise adjustment of hydrogel composition, maintaining this precision during scale-up is difficult, leading to variability and reproducibility issues [213]. In particular, inconsistencies across batches of natural polymers, decellularized ECM materials, and cell-derived components can lead to performance fluctuations and create regulatory approval barriers.

In addition, incorporating multi-functionalization approaches often increases manufacturing complexity and regulatory burden. Hydrogels incorporating multiple drugs, GFs, extracellular vesicles, and nanomaterials may face challenges related to batch-to-batch reproducibility, sterilization, scalability, and cost-effective production. Furthermore, regulatory approval becomes increasingly complicated when a single product combines biomaterial, drug, biologic, and cellular components. Future research should therefore focus not only on maximizing biological functionality but also on simplifying design architectures, improving manufacturing robustness, and establishing standardized quality-control procedures.

Some commonly used standard techniques in the laboratory, such as gamma irradiation, steam autoclaving, and ethylene oxide treatment, often impose detrimental effects on hydrogel systems [214]. These methods can disrupt the delicate architecture of polymer networks, weaken crosslinking integrity, and trigger degradation pathways that ultimately compromise mechanical stability and functional performance. The challenge is further intensified in bioengineered constructs incorporating proteins or living cells, as these components are highly sensitive to thermal, chemical, and radiative stress, which can lead to denaturation, loss of bioactivity, or irreversible cellular damage. Such inherent incompatibility represents a major barrier to clinical translation, necessitating a strategic shift toward materials designed to withstand sterilization-induced stress or toward aseptic fabrication approaches that avoid harsh terminal sterilization altogether. While pre-sterilization filtration of individual components can preserve structural and biological integrity, it introduces additional procedural complexity. It requires stringent process control. Adopting automated fabrication techniques and strict compliance with good manufacturing practice guidelines carefully optimized strategies to ensure both functionality and safety in clinical hydrogel applications, all of which are technically demanding to implement [215].

Finally, the inclusion of bioactive components such as GFs, stem cells, exosomes, and advanced nanomaterials substantially increases manufacturing costs and complicates large-scale production [216]. For these therapies to achieve widespread clinical adoption, particularly in resource-limited settings where burn injuries are highly prevalent, affordability must be addressed alongside therapeutic effectiveness and accessibility. Maintaining an appropriate balance between clinical performance and economic feasibility is therefore essential to ensure equitable use. In this context, small-molecule functionalization and scalable synthetic polymer platforms present more cost-efficient and practical alternatives to complex, biologically derived systems, without significantly compromising therapeutic potential. Moreover, the convergence of advanced engineering strategies with proactive regulatory planning will be critical to facilitate the integration of these innovative biomaterials into routine clinical practice.

### Advancing with emerging technology

Beyond improving biological functionality, future hydrogel development is expected to increasingly integrate AI, machine learning, and advanced biofabrication technologies. AI-assisted biomaterial design can accelerate the discovery and optimization of hydrogel formulations by predicting relationships between material composition, mechanical properties, degradation behavior, drug-release kinetics, and biological performance. Such data-driven approaches may reduce experimental workload and facilitate the development of multifunctional hydrogel systems tailored to specific pathological requirements. In parallel, advances in 3D printing and bioprinting technologies enable the fabrication of patient-specific hydrogel dressings with customized geometry, porosity, drug distribution, and mechanical properties that match the characteristics of individual burn wounds. Future personalized hydrogel platforms may further integrate biosensors, smart monitoring systems, and responsive drug-delivery mechanisms that adapt treatment to the dynamic wound microenvironment [16,217]. The convergence of biomaterials science, AI, digital health technologies, and precision medicine is expected to drive the development of next-generation hydrogel therapies for burn wound management. Furthermore, integrating patient-derived cells, extracellular vesicles, and personalized bioactive formulations with AI-guided design and bioprinting technologies may enable precision burn therapies that optimize healing outcomes while minimizing fibrosis and scar formation.

## Conclusion

Burn wound healing remains a significant clinical challenge because of the complex and dynamic pathological microenvironment. Conventional hydrogel dressings provide important physical benefits, including moisture retention, protection, and exudate management; however, their limited biological activity often restricts their therapeutic effectiveness in severe burn injuries. The emergence of hydrogel functionalization strategies has transformed these materials from passive wound coverings into bioactive therapeutic platforms that can actively modulate multiple stages of the healing process.

We highlight how incorporating peptides, proteins, GFs, bioactive small molecules, inorganic nanomaterials, ECM components, cells, and cell-derived vesicles enables hydrogels to address specific pathological barriers in burn repair. Importantly, the therapeutic benefits of these systems are determined not only by the incorporated bioactive agents but also by their interactions with the hydrogel matrix, which governs protection, retention, localization, and release within the wound microenvironment. Consequently, successful burn wound treatment increasingly depends on the rational integration of biomaterial properties with biological mechanisms rather than on the individual performance of isolated therapeutic components.

A central insight emerging from current research is that burn healing requires coordinated regulation of multiple biological processes rather than the enhancement of a single function. The most effective hydrogel systems are those that balance antimicrobial activity, immune modulation, angiogenesis, tissue regeneration, and matrix remodeling in a manner that aligns with the evolving physiological requirements of the wound. Therefore, the future clinical success of functionalized hydrogels will depend not only on increasing material complexity but also on achieving precise biological coordination, reproducible performance, and therapeutic relevance within the dynamic burn wound environment. Collectively, functionalized hydrogels represent a powerful platform for advancing regenerative burn care and provide an important foundation for the development of next-generation biomaterials that promote more effective, clinically meaningful tissue repair.

## Data Availability

No new data were created or analyzed in this study.
