# Supplementary material for: Advanced Hydrogel Functionalization Strategies for Burn-Related Wound Treatment
Source: Biomater Res. 2026 Sep 28;30:0406. doi: 10.34133/bmr.0406 (PMC13617242; doi:10.34133/bmr.0406)
Supplement: Supplementary file 1 [file bmr.0406.f1.docx]

**ADVANCED HYDROGEL FUNCTIONALIZATION STRATEGIES FOR BURN-RELATED WOUND TREATMENT**

Md Lemon Hasan^1^, Mohammad Faysal Al Mazid^2^, Yeasin Khan^3^, Al Basir^4^, Akhmad Irhas Robby^5^, Shihab Uddin^5,6^, Khandoker Asiqur Rahaman^5*^, Yoon Ki Joung ^1,7,8,*^

*^1^Biomaterials Research Center, Korea Institute of Science and Technology (KIST), Seoul 02792, Republic of Korea*

*^2^Department of Surgery, College of Medicine, Yonsei University, Seoul 06273, South Korea*

*^3^Department of Chemistry, Kyung Hee University, Seoul 02447, Republic of Korea.*

*^4^Interdisciplinary Research Center for Advanced Materials, King Fahd University of Petroleum and Minerals, Dhahran, 31261, Saudi Arabia*

*^5^Interdisciplinary Research Center for Bio Systems and Machines, King Fahd University of Petroleum & Minerals, Dhahran, 31261, Saudi Arabia*

*^6^Department of Bioengineering, King Fahd University of Petroleum & Minerals, Dhahran, 31261, Saudi Arabia*

*^7^Division of Bio-Medical Science & Technology, University of Science and Technology (UST), 113 Gwahangno, Yuseong-gu, Daejeon 34113, Republic of Korea*

*^8^KHU-KIST Department of Conversing Science and Technology, Kyung Hee University, Seoul, Republic of Korea*

****Address correspondence to:*** Khandoker Asiqur Rahaman; [khandoker.rahaman@kfupm.edu.sa](mailto:khandoker.rahaman@kfupm.edu.sa) and Yoon Ki Joung; ykjoung@kist.re.kr

**Supplementary figures**

**Fig. S1 (**A) Schematic representation of the pro-angiogenic hydrogel dressing developed from a hyperbranched polymer of polyPEGDA, a thiolated hyaluronic acid (HA-SH), and a cysteine-containing PR1P (cRP1P) peptide through Michael-type addition. (B) Photos of the gelling solution and fully cross-linked PH-PR1P hydrogel. (C) Quantitative analysis of loaded VEGF within hydrogels, determined by an ELISA kit. (D) Representative images of the tube formation assay using HUVECs. Quantitative analysis of the capillary length and the number of branch points in the tubule network. (E) Representative photos at each time point exhibit the process of wound closure. (F) Representative images of Masson's trichrome staining and collagen staining (green) of healed wounds. The slides are counterstained with DAPI (blue) to mark the nucleus, and scale bars are 50 μm. (G) Representative fluorescent images of CD31+ endothelial cells (green) show vascularization in healed wounds on day 14. The slides are counterstained with DAPI (blue) to mark the nucleus, and scale bars are 200 μm. (H) The stereological analysis of the vasculature length and surface area (n = 8). (I) Representative Western Blotting images and quantitation of pCaMKKβ/CaMKKβ ratio (n = 6). (J) Representative Western Blotting images and quantitation of p-AMPKβ/AMPKβ ratio (n = 6) (91). Copyright 2025, ACS.

**Fig. S2** (A) Schematic illustration of hybrid CSDP hydrogel preparation methods; CSDP-PACT simultaneously ensures bacterial clearance and enhances tissue regeneration in the burn model, greatly improving the Bioavailability of DVDMS/bFGF. (B) Antibacterial mechanisms of CSDP-PACT. (A) CSDP hydrogel promoted DVDMS release at lower pH values in wound areas, thereby enhancing DVDMS accumulation in bacteria. SEM, AFM, and confocal laser scanning microscopy (CLSM) images showed that DVDMS-PACT could destroy bacterial membranes and biofilms. (C) SEM images of MDR-S. aureus biofilms showing Anti-biofilm effects of CSDP-PACT after different treatments of light irradiation. Scale bar = 10 μm. Quantitative analysis of biofilm thickness was performed from confocal images, and crystal violet staining was used to quantify biofilm biomass after different treatments. (D) The multi-functional hydrogel was applied on days 1, 3, 5, 7, 10, and 14 to treat burn infections. The corresponding wound photographs showing the effect of DVDMS/bFGF nanohybrids hydrogel. Wound size is measured as a percentage of the initial wound size at day 0. The bacterial counts of MDR-S. aureus with hydrogel treatment in burn wounds at different times was also evaluated (140).

**Fig. S3** (A) Schematic representation of the formulation of ASC encapsulated conformable hydrogel. (B) ASCs loaded hydrogel applied in murine burn showed cell retention by bioluminescence imaging of the luciferase+ ASCs delivered within hydrogels (“hydrogel + cells”) or delivered by local injection (“cells alone”). (C)) Representative photos at each time point exhibit the process of wound closure. (D) The average healing time confirms that the hydrogel-ASC treatment accelerates healing compared with the control groups. (mean ± SD, n = 10, *P < 0.05) (92). (E) The formation of zwitterionic polysaccharide-based hydrogel. Loaded with adipose-derived stem cells (164), (F) Schematic illustration of adipose-derived stem cell spheroid-loaded enzyme-crosslinked gelatin hydrogel preparation (165).

**Fig. S4** (A) Schematic illustration showing the formulation of PP powder and PP hydrogel. (B) Optical photographs of the synthesis process of PP powder. (C) Representing photographs of PP and Cur-Mg@PP, ensuring hydrogel formation, where incorporation of curcumin in the Cur-Mg@PP hydrogel takes on a light color. (D) Representing photography, ensuring adhesion and self-healing properties of PP and Cur-Mg@PP hydrogels. Antimicrobial activity of different types of hydrogel formulation was evaluated on (E) E. coli, (F) P. aeruginosa, and (G) S. aureus in the disk diffusion assay. (H)Quantified diameters of bacteriostatic zones in the disk diffusion assay for 48, 40, and 56 h (97).
